# Supplementary material for: Piggy: a rapid, large-scale pan-genome analysis tool for intergenic regions in bacteria
Source: Gigascience. 2018 Mar 4;7(4):giy015. doi: 10.1093/gigascience/giy015 (PMC5890482; doi:10.1093/gigascience/giy015)
Supplement: GIGA-D-17-00244_Revision_1.pdf [file giy015_giga-d-17-00244_revision_1.pdf]

GigaScience

Piggy: A Rapid, Large-Scale Pan-Genome Analysis Tool for Intergenic Regions in Bacteria

--Manuscript Draft--

**Manuscript Number:**	GIGA-D-17-00244R1
**Full Title:**	Piggy: A Rapid, Large-Scale Pan-Genome Analysis Tool for Intergenic Regions in Bacteria
**Article Type:**	Technical Note
**Funding Information:**	Medical Research Council (G1000803) — Not applicable<br>United Kingdom Clinical Research Collaboration Translational Infection Research Initiative — Not applicable
**Abstract:**	Despite overwhelming evidence that variation in intergenic regions (IGRs) in bacteria can directly influence phenotypes, most current approaches for analysing pan-genomes focus exclusively on protein-coding sequences. To address this we present Piggy, a novel pipeline that emulates Roary except that it is based only on IGRs. We demonstrate the use of Piggy for pan-genome analyses of Staphylococcus aureus and Escherichia coli using large genome datasets. For S. aureus, we show that highly divergent ("switched") IGRs are associated with differences in gene expression, and we establish a multi-locus reference database of IGR alleles (igMLST; implemented in BIGSdb). Piggy is available at https://github.com/harry-thorpe/piggy and registered with SciCrunch (RRID: SCR_015941).
**Corresponding Author:**	Edward Feil<br><br>UNITED KINGDOM
**Corresponding Author Secondary Information:**	
**Corresponding Author's Institution:**	
**Corresponding Author's Secondary Institution:**	
**First Author:**	Harry A. Thorpe
**First Author Secondary Information:**	
**Order of Authors:**	Harry A. Thorpe<br>Sion C. Bayliss<br>Samuel K. Sheppard<br>Edward Feil
**Order of Authors Secondary Information:**	
**Response to Reviewers:**	Reviewer reports:<br><br>Reviewer #1: Piggy represents a potentially valuable tool to the field of comparative genomics. In general, additional details on how the algorithm works would be helpful to understand the results.<br><br>RESPONSE: We thank the reviewer for recognizing the value of our approach – we have added additional details concerning the algorithm throughout the manuscript as requested.<br><br>P1,L16; bacteria "has" impacts<br><br>RESPONSE: P2,L35-36: This line now reads “variation in intergenic regions (IGRs) in bacteria can directly influence phenotypes”

Powered by Editorial Manager® and ProduXion Manager® from Aries Systems Corporation

P2.L9: Add references to this first line

RESPONSE: P2,L46: Added references: McInerney et al. 2017; Andreani et al. 2017

P2,L14: Relationship between pan-genome and core will differ greatly on the organism chosen

RESPONSE:We agree with this point and have added the following text:

P2,L59-62: “More generally, the relationship between the size of the core and accessory genomes varies between species. Broadly, ecological generalists have large accessory genomes, whilst more ecologically restricted species, such as endosymbionts, have much smaller accessory genomes (McInerney et al. 2017; Andreani et al. 2017).”

P2,L30-34: This is a run-on sentence and could be broken up to improve clarity

RESPONSE: P3,L67-70: This sentence now reads:

The increasing availability of datasets containing thousands of isolates thus offers an unprecedented opportunity for describing the genetic basis of bacterial adaptation, although the scale of these data presents serious logistic and conceptual challenges in terms of data management and analysis.

P3,L11: I have several problems with this statement about LS-BSR. What do you mean that it is no longer specific. Specific to what? Also, you mention that this reduced specificity is a by-product of pre-clustering, but the next sentence indicates that Roary also uses pre-clustering. Why wouldn't that also affect the results?

RESPONSE:P3,L74-77: We apologise for the confusion, and on reflection agree with the referee that the text was not reflective of the relative performance of the two methods. We have changed the text accordingly.

P3,L16-17: You mention that Roary is "more accurate than LS-BSR" and this is likely based on one comparison in the Roary paper. This was the result of one simulated dataset, using an unknown version of USEARCH and unknown parameters for alignment. To be safe, if you want to still report these results, I would mention that Roary was more accurate than LS-BSR using one simulated dataset, although the details remain unclear. You could safely remove this statement and not detract from the rest of your manuscript.

RESPONSE:P3,L74-77: Again, we completely agree with the referee and have modified the text accordingly.

P3,L39: Reference for "15% of the genome" statement?

RESPONSE:P3,L86: Added references: Ochman and Caro-Quintero 2016; McCutcheon and Moran 2011

P13,L4-6: What lengths of IGRs do you consider? Is there a minimum length? What do you do at the beginning and ends of draft contigs? More detail here would be very helpful.

RESPONSE:We have provided more detail in the text as requested:

P7,L204-206: IGRs at the edge of contigs are excluded by default, but when they are included (using the --edges flag) the missing information is denoted by NA, for example ‘Gene_1 NA NA’.

P7,L207-209: By default, only IGRs between 30-1000 bp in length are included by Piggy, though these lengths can be user-defined using the --size flag (minimum length = 30 bp).

Powered by Editorial Manager® and ProduXion Manager® from Aries Systems Corporation

P13,L27: What BLASTN parameters do you use to merge similar clusters?

P7,L218-219: More detail provided: BLASTN defaults, except -word_size = 10

P13,L27: What thresholds do you decide on for presence/absence?

P7,L219-221: Thresholds are provided by --len_id and --nuc_id, and these are used to produce clusters. Once the clusters have been produced, the gene presence information is simply a matrix of these clusters vs strains.

Fig S1: These trees look to be unrooted, but am unsure of why

RESPONSE:The phandango tool provides a visual comparison between the relatedness based on core genome variation with differences in gene content. The use of an outgroup to root the tree is not required for this.

Reviewer #2: The manuscript entitled: "Piggy: A Rapid, Large-Scale Pan-Genome Analysis Tool for Intergenic Regions in Bacteria" introduces the pipeline Piggy for the analysis of intergenic regions (IGRs). The authors correctly point out that current approaches in pan-genome analysis focus purely on genes. They present a pipeline to address the remaining parts of the genome.
Based on published RNA-seq data the manuscript highlights that especially for the analysis of gene expression the state of the intergenic region can be relevant and should be considered carefully.
Since the presented pipeline equals to a great extent the approach of the software Roary, the main contribution of this work is the identification of switched IGRs. In particular, the handling of differently annotated gene borders is solved in a clever way.
So far no standard file format for pangenomic data has established but the output format of Roary can be used by a bunch of analysis and visualization tools (panX, Phandango, FriPan).
It is thus reasonable to use this format for the output of Piggy.
Since for large parts of the intergenic regions in bacteria the function is unknown and most of these regions are very short, I am not sure how accurate the reconstruction of the "panIGRome" by Piggy currently is (see point 1. below).
However, before I can recommend accepting the manuscript there are some further points I would like to see addressed by the authors.:

Major points:

1. Intergenic regions in bacteria are usually much shorter than protein-coding sequences. Thus the clustering of these regions is potentially more vulnerable to wrongly aligned short sequences. Please add a part on the clustering performance to the manuscript.

RESPONSE:We thank the referee for this important point, and have spent considerable time addressing this issue in detail. Additional analyses on clustering performance are incorporated in the text (in both the Methods, P6,178-187, Results, P8-9,L252-271, and Discussion, P14,L445-458) as described below, and we feel this significantly improves the paper.

Our approach to examining clustering performance was based on truncating IGRs and re-clustering them with the original set of IGRs. This was based on the logic that if the truncation had no effect (i.e. if the same clusters were recovered), then this provides reassurance that the clustering is not confounded by the length of the sequences, at least within the relevant parameters we are using.

This approach confirmed that 20-30 bp represents a minimum length for reliable clustering of IGRs for S. aureus, but possible slightly longer for E. coli. The incorrect clustering at these lengths was mostly driven by IGRs which are homologous to other IGRs over part, but not all of the sequence (as a result of rearrangements, HGT etc). In these cases when the IGR was truncated it could align equally well with multiple original IGR sequences, depending on which section of the sequence was retained during truncation. This may be a problem at the edge of contigs, but these IGRs are (now) removed by default (updated in the newest version of Piggy on GitHub) -

Powered by Editorial Manager® and ProduXion Manager® from Aries Systems Corporation

P7,L204-206. Due to the high number of incorrectly clustered IGRs when truncated to 10 bp, we recommend that these sequences are not included in the analysis at all.

2. page 16 line 27-39. Why did you use two different clusterings? One very loose clustering for Fig 2 and 3 and one more rigid for the rest of the manuscript? I do not see the point of using two different clusterings. Either two IGRs have the same origin or not. There should be an optimal value for --len_id where the clustering is close to the true relationship. And this one should be used for all subsequent analyses.

RESPONSE:With respect, we feel that there is no true --len_id which is appropriate for all situations, in the same way that there is no true --nuc_id. Of course it is true that either IGRs have the same origin or not, but when faced with real data the rules for assigning clusters are essentially pragmatic rather than grounded in biological certainties. Hence Piggy (and Roary, LS-BSR, PanOCT) use thresholds to define clusters. An IGR may acquire a deletion in one strain which means it is no longer the same length as the same IGR in other strains, despite sharing a common history.

The loose setting (--len_id 10) was used to enable a fair comparison with Roary results, where genes of different lengths are frequently clustered together. These can be the result of genuine truncations or assembly errors. Roary only requires that genes are >120 bp in length, and does not require genes to be similar in length in order to cluster together (fully explained on P5-6,L152-168). The stricter setting (--len_id 90) was used to detect switching, as this enables downstream filtering based on either length or nucleotide identity (P6,L166-168).

3. The text emphasizes that it is so far unknown whether genes and IGRs should be considered as independent or closely linked units. Likely this will depend on the context of the scientific question. Instead of separate genes g or IGRs i the set of both (i,g) can be considered. In this case one could get a first impression on the linkage of both. While the identification of switched IGRs in the manuscript uses the information of the flanking genes, I would have loved to read a bit more about this link in the two data examples. How many core genes are flanked by core IGRs? How many different genes can be found next to the same IGR and how many different IGR does a gene have? Even a first impression on these numbers would improve the quality of the manuscript.

RESPONSE:We agree that this is an important consideration, and so have done an analysis which is designed to be a first impression on these numbers. We analysed the number of core and accessory genes which are immediately upstream of core and accessory IGRs, and presented these data in a table (Table 2), and also in the text:

RESPONSE:P10,L302-312: We used the output of Piggy to investigate the degree of linkage between genes and IGRs. We identified all genomic loci consisting of an IGR flanked by two genes, and from these we identified all pairs of genes and IGRs where the IGR was upstream of the gene. We then grouped these according to whether the gene or IGR was core or accessory (Table 2). For the S. aureus ST22 data, 99.5% of core genes were immediately downstream of a core IGR, and 92.9% of the accessory genes were similarly downstream of an accessory IGR. When considering the wider S. aureus dataset the figures were similar; 92.6% of core genes were downstream of a core IGR, and 96.8% of accessory genes were downstream of an accessory IGR. Thus, the assignment of an IGR as core or accessory is strongly predictive of the corresponding assignment of the cognate downstream gene, which in turn points to strong background linkage between genes in IGRs in the genome.

P10,L324-327: There was tight linkage between genes and IGRs, with 97.9% of core genes being immediately downstream of core IGRs and 97.3% of accessory genes being similarly downstream of accessory IGRs; these results are consistent with those from S. aureus (Table 2).

In addition, please state how you proceeded with genes where a gene has an IGR > 30bp in one strain and an IGR < 30bp in another strain. Are those genes excluded from your analysis?

RESPONSE:When an IGR was > 30 bp in one strain and < 30 bp in another, then

Powered by Editorial Manager® and ProduXion Manager® from Aries Systems Corporation

	those sequences > 30 bp would be included and the others would not. This is because the IGRs are selected before the clustering is done, and so the relationships between these sequences is not known.<br><br>4. The pan-genome can be studied at all levels of divergence from the level of single lineages within pathogenic strains up to the level of all bacteria. Piggy has been demonstrated in two closely related datasets based on a single lineage from S. aureus and E. coli, respectively. I am wondering if this is the envisaged distance of genomes to analyze and whether the pipeline can be used on more diverse datasets. In the former case, the manuscript should state more precisely that piggy is intended only for closely related bacterial strains. In the latter case, I would like to see the addition of some further more distantly related strains of S. aureus and/or E. coli.<br><br>RESPONSE:We have now included an additional analysis consisting of a diverse collection of 1500 S. aureus isolates (P9,L294, Fig 2b). This clearly shows that the size of the species-wide S. aureus pan-genome is much greater than that of ST22 (fourfold increase in the number of accessory genes, and fivefold increase in accessory IGRs) (Table 1). There was also a corresponding decrease in the number of core elements, although this was much more modest. That Piggy identified >2000 core genes and >1000 core IGRs suggests that Piggy can cope with diverse datasets (Table 1).<br><br>5. paragraph starting at page 9 at line 44:<br>In this paragraph a resampling method is used to show that between certain strains of S. aureus genes linked to a switched IGR are on average more differentially expressed than other genes.<br>While the resampling approach is appropriate to produce p-values in this setting, I do not understand how these p-values have been adjusted. The Benjamini-Hochberg method is usually not used to change p-values, and one has to choose an acceptable false discovery rate. Which FDR did you choose? In addition, the observations need to be independent, which is clearly not the case in the 12 pairwise comparisons.<br>I would recommend to either just show the simulated p-values and choose a level of significance below 0.05 or explain much more detailed what has been adjusted and why.<br>In addition, please stick to lowercase "p" for the p-value. Also in Figure 4.<br><br>RESPONSE:P12,L384-393: The p-values have been left unadjusted, and those < 0.05 were deemed significant. Lowercase p was used throughout. “Independently” has been removed from the text.<br><br>6. I understand that the data provided by Piggy can be directly used to create an allele scheme. But I do not see the benefit of creating an allele scheme for IGRs compared to the wgMLST schemes. Could you please clarify how this scheme could be used and what would be the advantage compared to MLST, rMLST and wgMLST?<br><br>RESPONSE:The IGR scheme is not expected to be used in isolation, but rather can be combined with a scheme based on genes which may offer increased resolution in very closely related sets of strains. We have added some explanation of this:<br><br>P13,L421-424: “Although we do not expect a typing scheme based solely on IGRs to be widely used, supplementing protein-coding regions with IGR alleles may provide additional information regarding links between genotype and phenotype, as well as increased epidemiological and phylogenetic resolution.”<br><br>Minor issues:<br><br>Please explain more clearly why IGRs < 30 bp are excluded. Is this due to problems with the clustering and how did you determine the border at 30 bp?<br><br>RESPONSE:The exclusion of IGRs <30 bp is a conservative threshold as evidenced by the clustering assessment as described above.<br><br>Figure 1: The text in the flow diagram should be much larger.<br><br>RESPONSE:We have increased the size of the text in this figure.

Powered by Editorial Manager® and ProduXion Manager® from Aries Systems Corporation

	Figure 2: In my opinion accumulation curves in pan-genome studies are not very informative and could easily be replaced by a simple table with the average number per genome and the total number in the pan-genome. I suggest to replace Fig 2b and Fig 3b by such a table and use the opportunity to replace vague statements about the gradient and the plateau of the accumulation curve in the text. The accumulation curves could still appear in the supplemental material.<br><br>RESPONSE:Figures 2 and 3 have been merged into one (Figure 2), and the accumulation curves and vague statements have been removed. A new table (Table 1) has been created and the text adjusted.<br><br>Figure 4: You could highlight the points in Figure 4 corresponding to the genes from Figure 5<br><br>RESPONSE:Figure 5 only serves as an illustration of the data using some example genes. Highlighting these genes on Figure 4 may draw unnecessary attention to them, and this is not the message we are trying to convey, which is that there is a moderate and widespread effect of IGR divergence on gene expression which is not limited to a few hand-picked genes.
**Additional Information:**	
**Question**	**Response**
Are you submitting this manuscript to a special series or article collection?	No
**Experimental design and statistics**<br><br>Full details of the experimental design and statistical methods used should be given in the Methods section, as detailed in our Minimum Standards Reporting Checklist. Information essential to interpreting the data presented should be made available in the figure legends.<br><br>Have you included all the information requested in your manuscript?	Yes
**Resources**<br><br>A description of all resources used, including antibodies, cell lines, animals and software tools, with enough information to allow them to be uniquely identified, should be included in the Methods section. Authors are strongly encouraged to cite Research Resource Identifiers (RRIDs) for antibodies, model organisms and tools, where possible.<br><br>Have you included the information requested as detailed in our Minimum Standards Reporting Checklist?	Yes
**Availability of data and materials**	Yes

All datasets and code on which the conclusions of the paper rely must be either included in your submission or deposited in publicly available repositories (where available and ethically appropriate), referencing such data using a unique identifier in the references and in the “Availability of Data and Materials” section of your manuscript.<br><br>Have you have met the above requirement as detailed in our Minimum Standards Reporting Checklist?	

Manuscript Click here to download Manuscript Thorpe_gigasc_revised.docx

Piggy: A Rapid, Large-Scale Pan-Genome Analysis Tool for Intergenic Regions in Bacteria.

Harry A. Thorpe[1], Sion C. Bayliss[1], Samuel K. Sheppard[1], Edward J. Feil[1*]

[1]The Milner Centre for Evolution, Department of Biology and Biochemistry, University of Bath, Bath BA2 7AY.

*Corresponding author

Keywords: Pan-genome, Accessory genome, Genomics, Whole-genome sequencing (WGS), Bacteria, Intergenic Regions, igMLST, Gene Expression, *Staphylococcus aureus*, *Escherichia coli*.

Abstract

Despite overwhelming evidence that variation in intergenic regions (IGRs) in bacteria can directly influence phenotypes, most current approaches for analysing pan-genomes focus exclusively on protein-coding sequences. To address this we present Piggy, a novel pipeline that emulates Roary except that it is based only on IGRs. We demonstrate the use of Piggy for pan-genome analyses of *Staphylococcus aureus* and *Escherichia coli* using large genome datasets. For *S. aureus,* we show that highly divergent ("switched") IGRs are associated with differences in gene expression, and we establish a multi-locus reference database of IGR alleles (igMLST; implemented in BIGSdb). Piggy is available at https://github.com/harry-thorpe/piggy and registered with SciCrunch (RRID: SCR_015941).

Background

Whole-genome sequencing has revealed that, in many bacteria, individual strains frequently recruit new genes from a seemingly endless genetic reservoir (McInerney, McNally, and O'Connell 2017; Andreani, Hesse, and Vos 2017). The total complement of genes observed across all strains, known as the pan-genome, often numbers tens of thousands, up to an order of magnitude more than the number of genes present in any single genome. In contrast, the "core-genome", which refers to the complement of genes present in all (or the vast majority) of sampled isolates, can be significantly smaller than the total number of genes in any given genome (Medini et al. 2005; Page et al. 2015). For example, a study of 328 *Klebsiella pneumoniae* isolates, each of which harbour 4-5,000 genes, revealed a pan-genome of 29,886 genes; only 1,888 (6.8%) of which were universally present (core) (Holt et al. 2015). Similarly, genome data for 228 *Escherichia coli* ST131 isolates revealed a pan-genome of 11,401 genes, of which 2,722 (23.9%) were core (McNally et al. 2016). The degree of gene content variation in the latter study is particularly striking as these isolates were all from the same sequence type (ST), thus show limited nucleotide divergence in core genes, and are descended from a recent common ancestor. More generally, the relationship between the size of the core and accessory genomes varies between species, with ecologically diverse species having large accessory genomes, and ecologically restricted species (such as endosymbionts) having small accessory genomes (McInerney, McNally, and O'Connell 2017; Andreani, Hesse, and Vos 2017).

There is growing recognition that the acquisition of new genes through horizontal gene transfer (HGT) has a central role in ecological adaptation (Vos et al. 2015). The emergence and spread

of antibiotic resistance, underpinned by the transfer of plasmids and other mobile genetic elements (MGEs), is a pertinent example. The increasing availability of datasets containing thousands of isolates thus offers an unprecedented opportunity for describing the genetic basis of bacterial adaptation, although the scale of these data presents serious logistic and conceptual challenges in terms of data management and analysis.

Pioneering pan-genome analysis tools, such as PanOCT and PGAP relied on all-vs-all BLAST comparisons between protein sequences, and scaled approximately quadratically with the number of isolates (Fouts et al. 2012; Zhao et al. 2012). LS-BSR introduced a pre-clustering step which substantially reduced the number of BLAST comparisons, enabling it to be feasibly run on thousands of samples (Sahl et al. 2014). More recently, the Roary pipeline has rapidly gained popularity for scalable, user-friendly, pan-genome characterisation (Page et al. 2015).

The concept of the pan-genome, as described above, places an exclusive emphasis on genes; or, more specifically, open reading frames with the potential to encode proteins. This gene-centric perspective has both shaped, and been shaped by, the bioinformatics tools developed to interrogate the pan-genome. For example, Roary works by taking individual protein-coding sequences, pre-defined using Prokka annotation (Seemann 2014), and assigning each to a single cluster of homologous sequences. This approach thus excludes non protein-coding intergenic regions (IGRs) which typically account for approximately 15% of the genome (Ochman and Caro-Quintero 2016; McCutcheon and Moran 2011). This is clearly problematic for downstream attempts to identify genotype-phenotype links, as IGRs contain many important regulatory elements including, but not limited to, promoters, terminators, non-coding RNAs, and regulatory binding sites. Moreover, we have recently shown that IGRs are subject to purifying selection in the core-genomes of diverse bacterial species, even when known major regulatory elements are excluded (Thorpe et al. 2017; Molina and Van Nimwegen 2008), and a recent study has shown that intergenic variation is positively selected during *Pseudomonas aeruginosa* infections (Khademi and Jelsbak 2017).

Given that variation in IGRs can have profound phenotypic consequences, it is timely to consider how best to incorporate these sequences into pan-genome analyses. A key question is the degree to which protein-coding genes, and their cognate regulatory elements, should be considered a single “unit”, both selectively (in terms of co-adaptation) and in terms of physical

linkage on the chromosome. If physical linkage is assumed to be highly robust, such that genes are mostly transferred along with their cognate IGRs, then in principle the definition of a “gene” could be expanded to include the upstream regulatory regions. On the other hand, if there is moderate or weak linkage between genes and IGRs, such that IGRs can occasionally transfer independently, then the purview of the pan-genome could be expanded to include the full complement of IGR alleles in addition to protein-coding sequences.

Consistent with the second model, which allows for independent transfer of IGRs, a landmark study demonstrated that *E. coli* genes can apparently be regulated by alternative IGRs that frequently share no sequence similarity to each other (Oren et al. 2014). Moreover, the distribution of these IGRs was incongruent with gene trees, suggesting that recombination can act to replace one IGR with another resulting in regulatory “switches”; a process they call horizontal regulatory transfer (HRT) (Oren et al. 2014). It is important to note here that the term “switching” refers only to the replacement of an IGR by a non-homologous or highly divergent variant sequence. It does not specify that the replacement IGR has a particular origin, and could therefore correspond to a transfer from elsewhere in the same genome, or from another isolate. It was also noted that conserved flanking genes may facilitate this process by providing localised regions of homology. IGR switches can be accompanied by differential gene expression (Oren et al. 2014), and may provide a mechanism to offset the fitness costs of harbouring plasmids and other MGEs (McNally et al. 2016), pointing to a central role for this process in adaptation.

Our current understanding of the evolutionary dynamics of IGRs in the context of bacterial pan-genome leave many open questions. Specifically, it is unclear how IGRs are distributed among isolates within bacterial populations, how commonly IGRs and their cognate genes are co-transferred, or how the frequency of HRT relates to different functional gene categories. A more complete understanding of bacterial adaptation clearly requires a careful consideration of gene presence/absence alongside gene regulation. Here we address this by introducing a new pipeline called Piggy which closely emulates and complements the established pan-genome analysis pipeline Roary (Page et al. 2015). Input and output files for Piggy and Roary use the same format, and run in a similar time on modest computing resources. Piggy provides a means to rapidly identify IGR switches, and more broadly the means to examine the role of horizontal transfer in shaping the bacterial regulome. We demonstrate the utility of Piggy using large

genome datasets for single lineages within two bacterial species, both of which are of high public health importance; *Staphylococcus aureus* and *Escherichia coli*. Conventional pan-genome analyses are applied to analyse and compare core and accessory IGRs/genes in these lineages. In *S. aureus* we show an association between IGR switching and changes in gene expression, and demonstrate proof-of-principle by establishing a multilocus IGR scheme, (igMLST) in BIGSdb (Jolley and Maiden 2010). Piggy is available at (https://github.com/harry-thorpe/piggy) under the GPLv3 licence.

Methods

Datasets

The *S. aureus* dataset was assembled from published genome sequences (Reuter et al. 2015) available at http://www.ebi.ac.uk/ena (study number ERP001012). The *S. aureus* RNA-seq data was previously published (Warne et al. 2016), and is available at (http://www.ebi.ac.uk/ena, study number ERP009279). This was supplemented with the corresponding reference genomes, HO_5096_0412: HE681097, MRSA252: BX571856, Newman: AP009351, S0385: AM990992, available at (www.ncbi.nlm.nih.gov). The *E. coli* ST131 dataset was also from a previously published study (McNally et al. 2016), and is available at (http://datadryad.org/resource/doi:10.5061/dryad.d7d71). All complete genomes and assemblies were annotated with Prokka (Seemann 2014).

Roary and Piggy parameter settings

Roary (Page et al. 2015) was run using default parameters except for the following: -e -n (to produce alignments with MAFFT (Katoh and Standley 2013)); -i 90 (lower amino acid identity than the default); -s (to keep paralogs together); -z (to keep intermediate files). Piggy was run using default parameters except for --len_id, which controls the percentage of IGR sequences which must share similarity in order to be clustered together. For the *S. aureus* and *E. coli* ST131 datasets, Piggy was run twice, once with --len_id 10 and once with --len_id 90. The former was used for the pan-genome comparisons between genes and IGRs (Fig 2) in order to be comparable with Roary. Using a low length identity (--len_id 10) enabled homologous sequences of varying lengths (for example a truncated sequence) to cluster together. Roary does not provide a similar setting, and only requires that sequences have a minimum length of 120 bp. Genes in the same clusters defined by Roary may vary considerably in length, either due to genuine truncations or assembly errors. A relaxed --len_id setting of 10 was therefore

used in Piggy to provide consistency with Roary and to ensure that homologous IGRs are not erroneously placed in different clusters. A --len_id setting of 90 was subsequently used whenever "switched" IGRs were detected, as this enabled sequences to be subsequently filtered by either nucleotide or length identity.

RNA-seq analysis

Two biological replicates for each isolate were analysed. Kallisto (Bray et al. 2016) was used to quantify transcripts (--kmer-size 31 and --bootstrap-samples 100), and Sleuth (Pimentel et al. 2017) was used to normalise and filter the counts produced by Kallisto. These counts were then $\log_{10}$ transformed, and major axis (MA) regression was performed. Rockhopper2 (Tjaden 2015) was used to produce an operon map for each strain by grouping adjacent genes with similar expression profiles together into operons.

Clustering performance

We examined the clustering performance of Piggy by producing truncated variants of IGRs of lengths 10, 15, 20, 30, 50 bp, and comparing how the lengths of the IGRs altered the resulting clustering. The IGRs were truncated from a random starting point in the sequence, and each length was analysed separately. From the starting pool of IGRs from 10 randomly selected isolates, 1000 IGRs were chosen and truncated. These truncated variants were then added to the pool of IGRs and Piggy was run on them. Clustering patterns based on the truncated and original IGRs were then compared, with truncated IGRs placed in the same cluster as their progenitor sequences being assigned as correctly clustered. This analysis was performed on both the *S. aureus* ST22 and *E. coli* ST131 datasets.

Statistical analysis

All statistical analysis was performed within R version 3.3.2 (https://www.r-project.org). All plotting was performed with ggplot2 (Wickham 2009).

Results

Overview of the Piggy pipeline

Fig 1a shows an overview of the Piggy pipeline. The first step is to run Roary, as the gene presence absence output file from Roary is used as an input for Piggy. Piggy is then run using the same annotated assemblies as Roary, specifically GFF3 format files such as those

produced by Prokka (Seemann 2014). Piggy extracts intergenic sequences (IGRs) from these files, and uses the flanking gene names and their orientations to name the IGRs (Fig 1b).

Each IGR name contains three pieces of information: the upstream gene, the downstream gene, and their relative orientations (CO - co-oriented, DP - double promoter, DT - double terminator). For example, the IGR "Gene_1 Gene_2 DP" is flanked by Gene_1 and Gene_2, which are both downstream of the IGR (i.e. they are transcribed in opposite directions). IGRs at the edge of contigs are excluded by default, but when they are included (using the --edges flag) the missing information is denoted by NA, for example "Gene_1 NA NA". Including the gene neighbourhood information gives context to the IGR and enables identification of "switched" IGRs. By default, only IGRs between 30-1000 bp in length are included by Piggy, though these lengths can be user-defined using the --size flag (minimum length = 30 bp). The IGRs are then clustered with CD-HIT (Fu et al. 2012) at user-defined identity thresholds (--nuc_id - nucleotide identity, --len_id - length identity). The nucleotide identity is defined as SNPs / aligned sites, and the length identity is defined as shared sites / alignment length. These two flags allow the user to set the level of stringency for clustering. For example, a conservative approach is to set high values for both nucleotide and length identity such that IGRs must be similar in both nucleotide and length identity to cluster together. By relaxing the length identify whilst maintaining a high nucleotide identity threshold, highly related sequences still cluster even if one is truncated. The longest sequence from each cluster is then used to perform an all-vs-all BLASTN search (Camacho et al. 2009). This is used to merge similar clusters (BLASTN defaults, except -word_size = 10), which did not cluster with CD-HIT. These clusters are then used to produce an IGR presence absence matrix ("IGR_presence_absence.csv"), in the same format as the gene presence absence matrix ("gene_presence_absence.csv") produced by Roary. Up until this point, the pipeline is very similar to Roary (Page et al. 2015).

Switched IGR detection

Piggy identifies "switched" IGRs using two methods. For both methods, the term "switch" refers to two or more divergent IGR sequences occupying the same locus as defined by flanking genes, but does not specify an origin for the divergent IGR sequences (Oren et al. 2014). The first method identifies adjacent genes on the same contig (gene-pairs), and searches for IGR clusters which lie between these gene-pairs (Fig 1c). Instances where multiple IGR clusters correspond to the same gene-pair are identified as candidate switched IGRs. The second

method identifies instances where multiple IGR clusters occupy a locus upstream of a single gene cluster. This is a less conservative approach as only one of the two genes flanking the IGR is taken into account, (Fig 1c). The gene-pair method is used by default as it controls against detecting "switching" (recombination) events that encompass more than a single IGR, for example, cases where a mobile element has inserted between two genes. However such cases remain relevant as the regulation of the downstream gene may still be affected.

To ensure that differences in gene annotation between isolates, specifically artifactual variation in the start and end points of each gene, are not erroneously assigned as switching events, the first and last 30 bp of each flanking gene are searched against the IGRs with BLASTN. Any matches from these searches indicate differences in annotation of gene borders (rather than genuine differences between the IGRs), and these sequences are disregarded. In order to confirm that they represent genuine switching events, candidate switched IGRs are searched against each other with BLASTN with low complexity filtering turned off (-dust no). If there is no significant match they are classed as "switched", and if there is a significant match they are aligned using MAFFT (Katoh and Standley 2013). The resulting alignment is then used to calculate nucleotide identity (SNPs / shared sites), and length identity (number of shared sites / alignment length). These values can then be used to define an appropriate threshold to identify "switched" IGRs. To aid this, Piggy calculates within-cluster divergences for both genes and IGRs, and these divergences can be used to calibrate Piggy with Roary.

Clustering performance

The shorter lengths of IGRs compared with genes poses potential problems for alignment accuracy. We tested the clustering performance of Piggy by producing truncated variants of IGRs, adding these to the total complement of IGRs in an analysis, and then recording whether the truncated IGRs were clustered with their untruncated counterparts (Methods). For *S. aureus* ST22, 82% of IGRs truncated to 10 bp clustered together with the corresponding full length sequences, but this figure increased to > 99% when the length of the truncated sequences was 20 -bp. (Fig S1a). A similar increase was observed for the *E. coli* ST131 data, although in this case 50 bp was required for the percentage of correct assignments to be > 99%. (Fig S1b).

An inspection of the incorrectly clustered sequences from both datasets revealed that their progenitor sequences shared high sequence similarity in parts of their sequence to other IGR

clusters, but no sequence similarity in other parts of the sequence. This resulted in separate clusters which shared high sequence homology over parts of their sequences. When these sequences were truncated to assess the clustering, if the truncated part of the sequence was selected, then it could align to either of these IGR clusters. In many cases these alignments were perfect matches, and so the IGR could not be unambiguously placed. This problem is likely to be a result of non-homologous breaks at the edge of HGT events, and this is consistent with greater clustering accuracy in *S. aureus* ST22 compared with *E. coli* ST131, where the latter has a much larger pan-genome.

Staphylococcus aureus

S. aureus is an important skin-associated bacterium which is commonly carried asymptomatically, but can also cause a wide range of infections from minor skin infections to fatal bacteraemias. It has a clonal population structure consisting of discrete lineages (Feil et al. 2003). Although the core genome is relatively stable, phenotypic variation (e.g. resistance profiles, virulence traits, and host preference) is associated with a more dynamic accessory genome and the horizontal transfer of MGEs, such as the SCC*mec* element which confers resistance to β-lactam antibiotics (Lindsay and Holden 2004).

S. aureus ST22 (EMRSA-15) is a clinically important hospital-acquired methicillin resistant strain which is common in the UK and is rapidly expanding elsewhere in Europe and globally (Holden et al. 2013). Previous work has shown that *S. aureus* ST22 is clonal and shows relatively little variation in gene content (Holden et al. 2013; Reuter et al. 2015). In order to compare the pan-genomes of *S. aureus* at different scales, we analysed a diverse dataset of 1552 isolates from many lineages, and a smaller dataset of 500 ST22 isolates subsampled from the larger dataset (Reuter et al. 2015). The size of the gene and IGR pan and core-genomes were compared by analysing both datasets with Roary and Piggy. Frequency histograms were plotted for both genes and IGRs (Fig 2a-b).

The gene-IGR frequency histogram for ST22 (Fig 2a) shows that there are 2,409 core genes and 1,556 core IGRs, where core is defined as gene presence in > 95% of isolates (Table 1). When the whole species is considered, these numbers drop to 2,129 and 1,134, respectively. The fact that there are fewer core IGRs than core genes is in part due to the exclusion of IGRs < 30 bp (many of which are intra-operonic), but also likely reflects faster evolution of IGRs. Both

distributions conform to the U-shape typically found in such analyses where the majority of genes/IGRs are either very common or very rare, however the distribution of genes and IGRs is shifted towards the rare sequences when the whole species is considered rather than only ST22.

We used the output of Piggy to investigate the degree of linkage between genes and IGRs. We identified all genomic loci consisting of an IGR flanked by two genes, and from these we identified all pairs of genes and IGRs where the IGR was upstream of the gene start. We then grouped these according to whether the gene or IGR was core or accessory (Table 2). For the *S. aureus* ST22 data, 99.5% of core genes were immediately downstream of a core IGR, and 92.9% of the accessory genes were similarly downstream of an accessory IGR. When considering the wider *S. aureus* dataset the figures were similar; 92.6% of core genes were downstream of a core IGR, and 96.8% of accessory genes were downstream of an accessory IGR. Thus, the assignment of an IGR as core or accessory is highly predictive of the corresponding assignment of the cognate downstream gene, which in turn points to strong background linkage between genes in IGRs in the genome.

***Escherichia coli* ST131**

The utility of Piggy was further validated by re-analysing data from a recent study on the widespread and clinically important *E. coli* lineage ST131 (McNally et al. 2016). This dataset contains 236 clinical *E. coli* ST131 isolates from human, domesticated animal, and avian hosts. *E. coli* is a more genetically diverse species than *S. aureus*, and unsurprisingly *E. coli* ST131 has a larger pan-genome than *S. aureus* ST22, with 12,806 genes and 16,429 IGRs (Fig 2c, Table 1). More surprisingly, *E. coli* ST131 has a larger pan-genome than the whole *S. aureus* species. Within *E. coli* ST131, 3,930 genes and 2,296 IGRs were core out of an average of 4,689 genes and 2,984 IGRs per isolate. Thus despite the differences between the two species in their level of diversity there was a consistent signal of a lower number of core IGRs than core genes, and a high number of accessory IGRs compared to accessory genes. There was tight linkage between genes and IGRs, with 97.9% of core genes being immediately downstream of core IGRs and 97.3% of accessory genes being similarly downstream of accessory IGRs; these results are consistent with those from *S. aureus* (Table 2).

The data from *S. aureus* and *E. coli* shows a background of strong linkage between genes and IGRs. However, this linkage is not perfect; some core genes are associated with accessory IGRs (and vice-versa), and the linkage is weaker over long timescales (across the whole *S. aureus* species compared to within ST22). Previous work has examined this linkage and found evidence of widespread IGR regulatory switching, where genes are regulated by alternative IGRs in different isolates (Oren et al. 2014). Piggy provides a list of candidate switching events together for both "gene-pair" and "upstream" approaches (see Methods) at different thresholds of nucleotide identity. For the *E. coli* ST131 data, the pipeline detected 61 cases of putative IGR switching using the most conservative settings (i.e. the conservative gene-pair method, and the alternative IGRs showing no sequence similarity by BLASTN). Relaxing the threshold of sequence identity to < 90% resulted in the identification of an additional 317 candidate switching events, though these possibly reflect either relaxed or positive selection.

Switched IGRs influence gene expression in *S. aureus*

To examine whether switches in IGRs affect the expression of cognate (downstream) genes, we used a previously published RNA-seq dataset based on four reference *S. aureus* isolates HO_5096_0412 (ST22), Newman (CC8), MRSA252 (CC36), and S0385 (CC398) (Warne et al. 2016). Each of these *S. aureus* references isolate represents a distinct major clonal complex, and all were grown under identical conditions with each experiment being replicated. Thus these data provide evidence of the natural variation in gene expression within the *S. aureus* population. By analysing these data alongside the output from Piggy, it is possible to test the extent to which IGR switches between these four genomes can account for the observed variation in gene expression between clonal complexes. First Roary was used to identify a set of 2094 single copy core genes present in all four isolates, and then expression of these core genes was quantified using Kallisto (Bray et al. 2016). To do this we used RNA-seq data for two replicates for each of the four reference genomes. The tpm (Transcripts per Kilobase Million) values for each gene are given in Table S1. We then used Sleuth (Pimentel et al. 2017) to normalise and filter these counts.

To check the consistency of the data between biological replicates, we first plotted two replicates for each isolate against each other (e.g. Newman replicate 1 vs Newman replicate 2) (Fig 3). These plots were tightly correlated (mean R^2 = 0.98), confirming that the expression values for individual genes were consistent between replicates. We then plotted between-isolate

comparisons, again using both replicates for each genome (e.g. Newman replicate 1 vs MRSA252 replicate 1, and Newman replicate 2 vs MRSA252 replicate 2) (Fig 3). These comparisons revealed considerably more scatter, with R^2 values ranging from 0.76 to 0.9. Given the extremely high R^2 values for within-isolate comparisons, the decrease in R^2 for between-isolate comparisons reflects genuine differences in expression between the isolates. We note that a small number of genes show very striking differences in expression between the clonal complexes. For example, the expression of *mepA*, which encodes a multidrug efflux pump, was ~250 fold higher in Newman compared with the other isolates.

The genomes of each pair of isolates were analysed using Roary and Piggy to identify switched IGRs with a nucleotide identity threshold of < 90% for IGR clusters. For each pair of isolates, we then identified all genes immediately downstream of a switched IGR. As a single switched IGR might impact on the expression of more than one co-transcribed downstream genes we also considered all genes linked in a single operon that could be impacted by a single switching event upstream affecting a shared promoter. For each pair of isolates, we thus identified all core genes putatively affected by upstream IGR switches. We then tested whether these genes showed a higher degree of differential expression by conducting Monte Carlo permutation tests on the residuals from the regressions (Fig 3). For each pairwise comparison of isolates, we summed the residuals of the genes with switched IGRs (shown as red points in Fig 3), and compared this to a distribution obtained by resampling (without replacement) 100,000 random sets of the same number of genes and summing their residuals. We computed a one-tailed p-value by dividing the number of permutations with summed residuals greater than the observed value by 100,000 (Fig 3). Because we used both replicates separately (e.g. Newman replicate 1 vs S0385 replicate 1, and Newman replicate 2 vs S0385 replicate 2), each comparison between pairs of isolates was tested twice. In 9/12 pairwise comparisons, the observed residuals of the genes downstream of switched IGRs were significantly ($p < 0.05$) greater than expected from the resampled data, indicating that genes with switched IGRs were more differentially expressed than those without. Of the three remaining comparisons, two corresponded to comparisons between HO_5096_0412 and S0385 ($p = 0.17$, and $p = 0.055$), and one between HO_5096_0412 and Newman ($p = 0.054$). The second comparison between HO_5096_0412 and Newman was the most weakly significant result ($p = 0.025$). Thus, the two replicates for each individual pairwise comparison were largely concordant with each other.

Our analysis confirms that genes downstream of switched IGRs are on average more likely to be differentially expressed than genes not associated with IGR switches as identified using Piggy. To illustrate the genomic context and expression differences of genes with switched IGRs, we selected three of the most differentially expressed genes with IGR switches for the Newman vs MRSA252 comparison, and plotted nucleotide identity across the IGR (calculated as a 20-bp sliding window) alongside gene expression (Fig 4).

Compatibility and scalability

We have so far demonstrated that Piggy can be used to analyse the intergenic component of the pan-genome and identify IGR switches, and shown that these switches have biological relevance with respect to gene expression. Importantly, Piggy is designed such that the output files are compatible with existing software and databases. The “IGR_presence_absence.csv” file has an identical format to the “gene_presence_absence.csv” file produced by Roary, and can be loaded directly into the interactive browser-based viewer phandango (Hadfield et al. 2017) (Fig S2). It can also be used as input, along with a traits file, to Scoary (Brynildsrud et al. 2016) to test for associations between IGRs and phenotypic traits. Moreover, the “representative_clusters_merged.fasta” file can be loaded directly into BIGSdb (Jolley and Maiden 2010) to create an allele scheme for IGRs. In order to provide proof-of-principle, we created a multilocus IGR (igMLST) scheme in BIGSdb. Briefly, 2631 unique IGR sequences with length ≥ 30bp, from 7 *S. aureus* reference genomes, were entered into the database locus list. Using functionality within the database, these sequences were grouped as a searchable scheme (S_aureus_Intergenic_PIGGY), comparable to MLST, rMLST and wgMLST schemes (Maiden et al. 2013; Jolley et al. 2012; Sheppard, Jolley, and Maiden 2012). The distribution of IGRs was analysed for all isolates in the database, identifying IGRs as present in the respective genome if a hit was recorded with nucleotide identity ≥ 70% over ≥ 50% of the sequence using a BLAST word size of 7 bp. The scheme can be found at https://sheppardlab.com/resources/. Although we do not expect a typing scheme based solely on IGRs to be widely used, supplementing protein-coding regions with IGR alleles may provide additional information regarding links between genotype and phenotype, as well as increased epidemiological and phylogenetic resolution.

Discussion

Whole-genome sequence datasets consisting of hundreds or even thousands of bacterial isolates have revealed pan-genomes of many thousands of genes and large differences in gene content between isolates of the same species. Currently, pan-genome diversity is considered almost exclusively in terms of protein-coding genes, despite overwhelming evidence that variation within IGRs impacts on phenotypes. Here we address this by introducing Piggy, a pipeline specifically designed to incorporate IGRs into routine pan-genome analyses by working in close conjunction with Roary (Page et al. 2015).

The utility of this approach is demonstrated using large datasets of *S. aureus* and *E. coli* ST131. Consistent with previous analyses of protein-coding regions (Holden et al. 2013; McNally et al. 2016), the IGR component of the ST131 pan-genome (the "panIGRome") is considerably larger than that for *S. aureus* ST22, and surprisingly is also larger than the pan-genome of the whole *S. aureus* species. There was more diversity within IGRs than genes in both species. While some IGRs may be essential for expression of multiple genes, IGRs are broadly subject to weaker purifying selection than protein coding genes (Thorpe et al. 2017). The maintenance of core IGRs in both bacterial genome datasets is consistent with selection acting to conserve them and allows alignment and analysis in much the same way as protein-coding regions.

The current exclusion of IGRs from routine pan-genome or cgMLST analyses may in part reflect perceived difficulties in the alignment and subsequent cluster definition, particularly if the sequences are very short. We therefore validated the pipeline by investigating clustering accuracy as a function of sequence length by truncating the IGR sequences and recording whether they remained in the same cluster as their full-length counterparts. For *S. aureus*, the data showed that truncated IGRs > 20 bp almost always remained in the original cluster, confirming that the minimum length permitted in the pipeline of 30-bp is conservative. For *E. coli*, truncating the sequences had greater impact on cluster assignments, and a minimum length of 50 bp would be a safer setting in this case. The problems with clustering shorter sequences in *E. coli*, compared to *S. aureus*, are not due to the length of the sequence *per se* but reflect the higher rate of recombination in this species. This means that the IGRs are more likely to be chimeric in structure, with localised regions within the IGRs showing a high level of homology to different clusters. This leads to cluster assignment being dependant not so much on length, but on which part of the truncated sequence happened to be retained.

Variation within regulatory elements located within IGRs can impact on the expression of the downstream gene (Oren et al. 2014). Piggy (alongside Roary) provides the means to combine information on genes and their cognate IGRs thus facilitating the detection of “switched” IGRs and downstream genes that are potentially affected. We have shown that in *S. aureus*, genes with switched upstream IGRs show a higher degree of differential expression than those without. This is consistent with previous work on *E. coli* (Oren et al. 2014), and suggests that the identification of IGR switches using Piggy can provide a useful indication of differential gene expression, even in the absence of RNA-seq data. However, we note that high divergence within IGRs does not necessarily imply selection for differential gene expression, and may instead simply reflect weaker selective constraints. A clear direction for future work is to make constructs consisting of genes with alternative IGRs, in order to directly measure the effect of natural IGR variants on gene expression. Similar experiments have previously been performed in *E. coli* based on variation within promoters (Shimada et al. 2014), and IGRs more broadly (Oren et al. 2014). The importance of changes in gene expression mediated by intergenic variation as a route of adaptation is currently unknown, but one recent study suggested that intergenic changes are strongly positively selected in *Pseudomonas aeruginosa* during infection in patients with cystic fibrosis, and more work is required to test the generality of these findings (Khademi and Jelsbak 2017).

Conclusions

Driven by recent technical advances in high-throughput sequencing, large whole-genome datasets have provided powerful evidence concerning the genetic determinants that underlie complex multifactorial phenotypes such as virulence. Moreover, associating variation in core and accessory genes with phenotype data is providing new fundamental insight into the ecology and evolution of bacteria. However, in much the same way that non-protein coding DNA in the human genome was initially dismissed as “junk”, omitting IGRs from bacterial genome analysis severely limits our ability to draw inferences on the regulation of gene expression and associated phenotypic consequences. By developing Piggy as an easy-to-use bioinformatics tool with output files that are compatible with existing software and databases (eg Roary, Phandango; Figure S1, Scoary, BIGSdb) we envisage that combined information from genes and their cognate IGRs will vastly improve our understanding of genome evolution in bacteria.

Declarations

Ethics approval and consent to participate: Not applicable

Consent for publication: Not applicable

Availability of data and material: The *S. aureus* ST22 dataset was assembled from published genome sequences of the clinically important lineage ST22 (EMRSA-15) (Reuter et al. 2015) available at http://www.ebi.ac.uk/ena (study number ERP001012). The *S. aureus* RNA-seq data was previously published (Warne et al. 2016), and is available at (http://www.ebi.ac.uk/ena, study number ERP009279). This was supplemented with the corresponding reference genomes, all available at (www.ncbi.nlm.nih.gov), HO_5096_0412: HE681097, MRSA252: BX571856, Newman: AP009351, S0385: AM990992. The *E. coli* ST131 dataset (McNally et al. 2016) is available at (http://datadryad.org/resource/doi:10.5061/dryad.d7d71).

Piggy is available at (https://github.com/harry-thorpe/piggy) under the GPLv3 licence.

Competing interests: Not applicable

Funding: The *Staphylococcus aureus* genome sequences were generated as part of a study supported by a grant from the United Kingdom Clinical Research Collaboration Translational Infection Research Initiative and the Medical Research Council (grant number G1000803, held by Sharon Peacock) with contributions from the Biotechnology and Biological Sciences Research Council; the National Institute for Health Research on behalf of the Department of Health; and the Chief Scientist Office of the Scottish Government Health Directorate, on which E.J.F. was a principal investigator and S.C.B. was a postdoctoral researcher. H.A.T. is funded by a University of Bath research studentship. The funders had no role in study design, data collection and analysis, decision to publish, or preparation of the manuscript. The authors declare that they have no competing interests.

Authors' contributions: HAT designed and implemented the pipeline, and carried out the majority of the analyses, with input from EJF, SCB and SKS. HAT and EJF wrote the manuscript with input from SKS and SCB.

Acknowledgements: We are very grateful to Torsten Seemann, Andrew Page and João Carriço for encouragement and helpful feedback. We are also grateful to Matt Holden for provision of the *S. aureus* RNA-seq data, to Sandra Reuter for help with the *S. aureus* ST22 data, and to Alan McNally for the *E. coli* ST131 data. This work also greatly benefitted from access to the Medical Research Council funded Cloud Infrastructure for Microbial Bioinformatics (MRC-CLIMB).

References

Andreani, Nadia Andrea, Elze Hesse, and Michiel Vos. 2017. "Prokaryote Genome Fluidity Is Dependent on Effective Population Size." *The ISME Journal* 11 (7):1719–21.

Bray, Nicolas L., Harold Pimentel, Páll Melsted, and Lior Pachter. 2016. "Near-Optimal Probabilistic RNA-Seq Quantification." *Nature Biotechnology* 34 (5):525–27.

Brynildsrud, Ola, Jon Bohlin, Lonneke Scheffer, and Vegard Eldholm. 2016. "Rapid Scoring of Genes in Microbial Pan-Genome-Wide Association Studies with Scoary." *Genome Biology* 17 (1):238.

Camacho, Christiam, George Coulouris, Vahram Avagyan, Ning Ma, Jason Papadopoulos, Kevin Bealer, and Thomas L. Madden. 2009. "BLAST+: Architecture and Applications." *BMC Bioinformatics* 10 (December):421.

Feil, Edward J., Jessica E. Cooper, Hajo Grundmann, D. Ashley Robinson, Mark C. Enright, Tony Berendt, Sharon J. Peacock, et al. 2003. "How Clonal Is Staphylococcus Aureus?" *Journal of Bacteriology* 185 (11):3307–16.

Fouts, Derrick E., Lauren Brinkac, Erin Beck, Jason Inman, and Granger Sutton. 2012. "PanOCT: Automated Clustering of Orthologs Using Conserved Gene Neighborhood for Pan-Genomic Analysis of Bacterial Strains and Closely Related Species." *Nucleic Acids Research* 40 (22):e172.

Fu, Limin, Beifang Niu, Zhengwei Zhu, Sitao Wu, and Weizhong Li. 2012. "CD-HIT: Accelerated for Clustering the next-Generation Sequencing Data." *Bioinformatics* 28 (23):3150–52.

Hadfield, James, Nicholas J. Croucher, Richard J. Goater, Khalil Abudahab, David M. Aanensen, and Simon R. Harris. 2017. "Phandango: An Interactive Viewer for Bacterial Population Genomics." *bioRxiv*. https://doi.org/10.1101/119545.

Holden, Matthew T. G., Li-Yang Hsu, Kevin Kurt, Lucy A. Weinert, Alison E. Mather, Simon R. Harris, Birgit Strommenger, et al. 2013. "A Genomic Portrait of the Emergence, Evolution, and Global Spread of a Methicillin-Resistant Staphylococcus Aureus Pandemic." *Genome Research* 23 (4):653–64.

Holt, Kathryn E., Heiman Wertheim, Ruth N. Zadoks, Stephen Baker, Chris A. Whitehouse, David Dance, Adam Jenney, et al. 2015. "Genomic Analysis of Diversity, Population Structure, Virulence, and Antimicrobial Resistance in Klebsiella Pneumoniae, an Urgent Threat to Public Health." *Proceedings of the National Academy of Sciences of the United States of America* 112 (27):E3574–81.

Jolley, Keith A., Carly M. Bliss, Julia S. Bennett, Holly B. Bratcher, Carina Brehony, Frances M. Colles, Helen Wimalarathna, et al. 2012. "Ribosomal Multilocus Sequence Typing: Universal Characterization of Bacteria from Domain to Strain." *Microbiology* 158 (Pt 4):1005–15.

Jolley, Keith A., and Martin C. J. Maiden. 2010. "BIGSdb: Scalable Analysis of Bacterial Genome Variation at the Population Level." *BMC Bioinformatics* 11 (1):595.

Katoh, Kazutaka, and Daron M. Standley. 2013. "MAFFT Multiple Sequence Alignment Software Version 7: Improvements in Performance and Usability." *Molecular Biology and Evolution* 30 (4):772–80.

Khademi, Hossein, and Lars Jelsbak. 2017. "Host Adaptation Mediated by Intergenic Evolution in a Bacterial Pathogen." *bioRxiv*. https://doi.org/10.1101/236000.

Lindsay, Jodi A., and Matthew T. G. Holden. 2004. "Staphylococcus Aureus: Superbug, Super Genome?" *Trends in Microbiology* 12 (8):378–85.

Maiden, Martin C. J., Melissa J. Jansen van Rensburg, James E. Bray, Sarah G. Earle,

Suzanne A. Ford, Keith A. Jolley, and Noel D. McCarthy. 2013. “MLST Revisited: The Gene-by-Gene Approach to Bacterial Genomics.” *Nature Reviews. Microbiology* 11 (10):728–36.

McCutcheon, John P., and Nancy A. Moran. 2011. “Extreme Genome Reduction in Symbiotic Bacteria.” *Nature Reviews. Microbiology* 10 (1):13–26.

McInerney, James O., Alan McNally, and Mary J. O’Connell. 2017. “Why Prokaryotes Have Pangenomes.” *Nature Microbiology* 2 (March):17040.

McNally, Alan, Yaara Oren, Darren Kelly, Ben Pascoe, Steven Dunn, Tristan Sreecharan, Minna Vehkala, et al. 2016. “Combined Analysis of Variation in Core, Accessory and Regulatory Genome Regions Provides a Super-Resolution View into the Evolution of Bacterial Populations.” *PLoS Genetics* 12 (9):e1006280.

Medini, Duccio, Claudio Donati, Hervé Tettelin, Vega Masignani, and Rino Rappuoli. 2005. “The Microbial Pan-Genome.” *Current Opinion in Genetics & Development* 15 (6):589–94.

Molina, Nacho, and Erik Van Nimwegen. 2008. “Universal Patterns of Purifying Selection at Noncoding Positions in Bacteria.” *Genome Research* 18 (1):148–60.

Ochman, H., and A. Caro-Quintero. 2016. “Genome Size and Structure, Bacterial.” In *Encyclopedia of Evolutionary Biology*, edited by Richard M. Kliman, 179–85. Oxford: Academic Press.

Oren, Yaara, Mark B. Smith, Nathan I. Johns, Millie Kaplan Zeevi, Dvora Biran, Eliora Z. Ron, Jukka Corander, Harris H. Wang, Eric J. Alm, and Tal Pupko. 2014. “Transfer of Noncoding DNA Drives Regulatory Rewiring in Bacteria.” *Proceedings of the National Academy of Sciences of the United States of America* 111 (45):16112–17.

Page, Andrew J., Carla A. Cummins, Martin Hunt, Vanessa K. Wong, Sandra Reuter, Matthew T. G. Holden, Maria Fookes, Daniel Falush, Jacqueline A. Keane, and Julian Parkhill. 2015. “Roary: Rapid Large-Scale Prokaryote Pan Genome Analysis.” *Bioinformatics* 31 (22):3691–93.

Pimentel, Harold, Nicolas L. Bray, Suzette Puente, Páll Melsted, and Lior Pachter. 2017. “Differential Analysis of RNA-Seq Incorporating Quantification Uncertainty.” *Nature Methods* 14 (7):687–90.

Reuter, Sandra, Estee M. Török, Matthew T. G. Holden, Rosy Reynolds, Kathy E. Raven, Beth Blane, Tjibbe Donker, et al. 2015. “Building a Genomic Framework for Prospective MRSA Surveillance in the United Kingdom and the Republic of Ireland.” *Genome Research*, December. https://doi.org/10.1101/gr.196709.115.

Sahl, Jason W., J. Gregory Caporaso, David A. Rasko, and Paul Keim. 2014. “The Large-Scale Blast Score Ratio (LS-BSR) Pipeline: A Method to Rapidly Compare Genetic Content between Bacterial Genomes.” *PeerJ* 2 (April):e332.

Seemann, Torsten. 2014. “Prokka: Rapid Prokaryotic Genome Annotation.” *Bioinformatics* 30 (14):2068–69.

Sheppard, Samuel K., Keith A. Jolley, and Martin C. J. Maiden. 2012. “A Gene-by-Gene Approach to Bacterial Population Genomics: Whole Genome MLST of Campylobacter.” *Genes* 3 (2):261–77.

Shimada, Tomohiro, Yukiko Yamazaki, Kan Tanaka, and Akira Ishihama. 2014. “The Whole Set of Constitutive Promoters Recognized by RNA Polymerase RpoD Holoenzyme of Escherichia Coli.” *PloS One* 9 (3):e90447.

Thorpe, Harry A., Sion C. Bayliss, Laurence D. Hurst, and Edward J. Feil. 2017. “Comparative Analyses of Selection Operating on Non-Translated Intergenic Regions of Diverse Bacterial Species.” *Genetics*, March. https://doi.org/10.1534/genetics.116.195784.

Tjaden, Brian. 2015. “De Novo Assembly of Bacterial Transcriptomes from RNA-Seq Data.” *Genome Biology* 16 (January):1.

Vos, Michiel, Matthijn C. Hesselman, Tim A. te Beek, Mark W. J. van Passel, and Adam Eyre-Walker. 2015. “Rates of Lateral Gene Transfer in Prokaryotes: High but Why?” *Trends in Microbiology* 23 (10):598–605.

Warne, Ben, Catriona P. Harkins, Simon R. Harris, Alexandra Vatsiou, Nicola Stanley-Wall, Julian Parkhill, Sharon J. Peacock, Tracy Palmer, and Matthew T. G. Holden. 2016. “The Ess/Type VII Secretion System of Staphylococcus Aureus Shows Unexpected Genetic Diversity.” *BMC Genomics* 17 (March):222.

Wickham, Hadley. 2009. *Ggplot2: Elegant Graphics for Data Analysis*. 2nd ed. Springer Publishing Company, Incorporated.

Zhao, Yongbing, Jiayan Wu, Junhui Yang, Shixiang Sun, Jingfa Xiao, and Jun Yu. 2012. “PGAP: Pan-Genomes Analysis Pipeline.” *Bioinformatics* 28 (3):416–18.

Figures

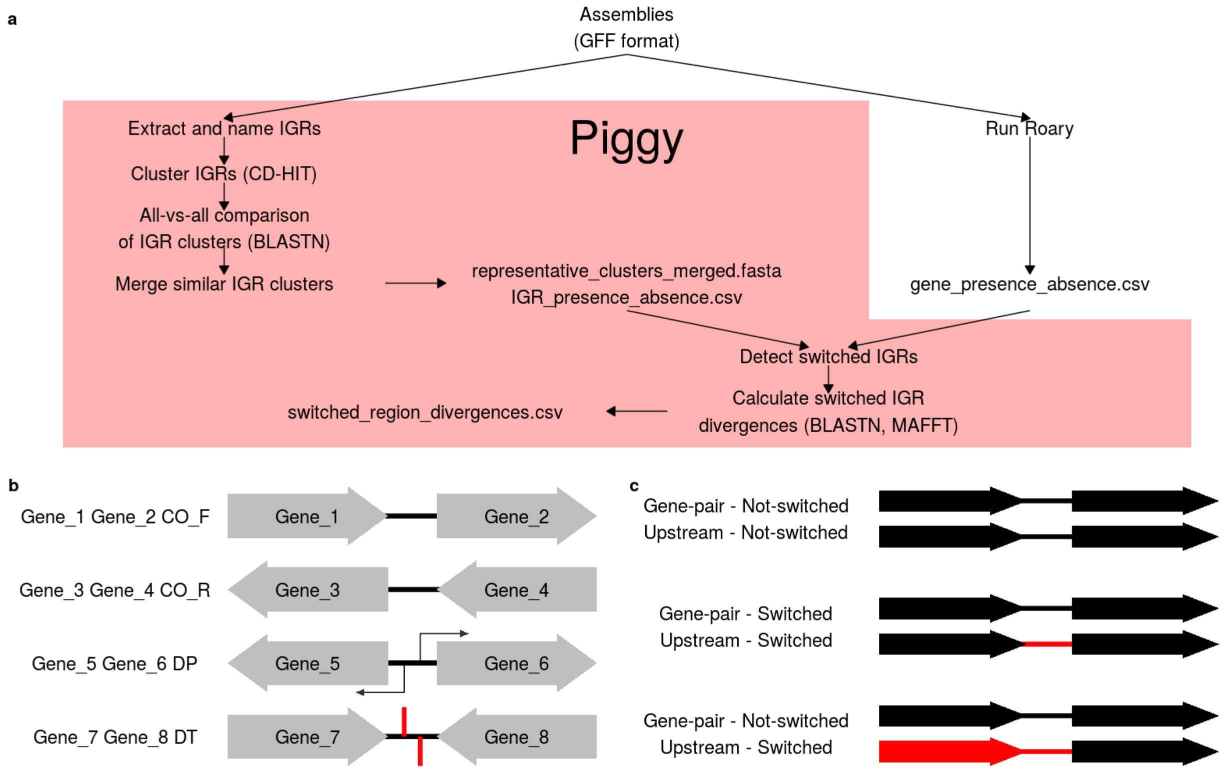

Fig 1: An overview of the Piggy pipeline. a) A schematic to illustrate the Piggy pipeline and how it works alongside Roary. b) IGRs are named according to their flanking genes and their orientations. This naming scheme enables Piggy to link genes with their associated IGRs, and provides information on their orientations. c) A schematic to illustrate the difference between the “gene-pair” and “upstream” methods used to identify candidate switched IGRs.

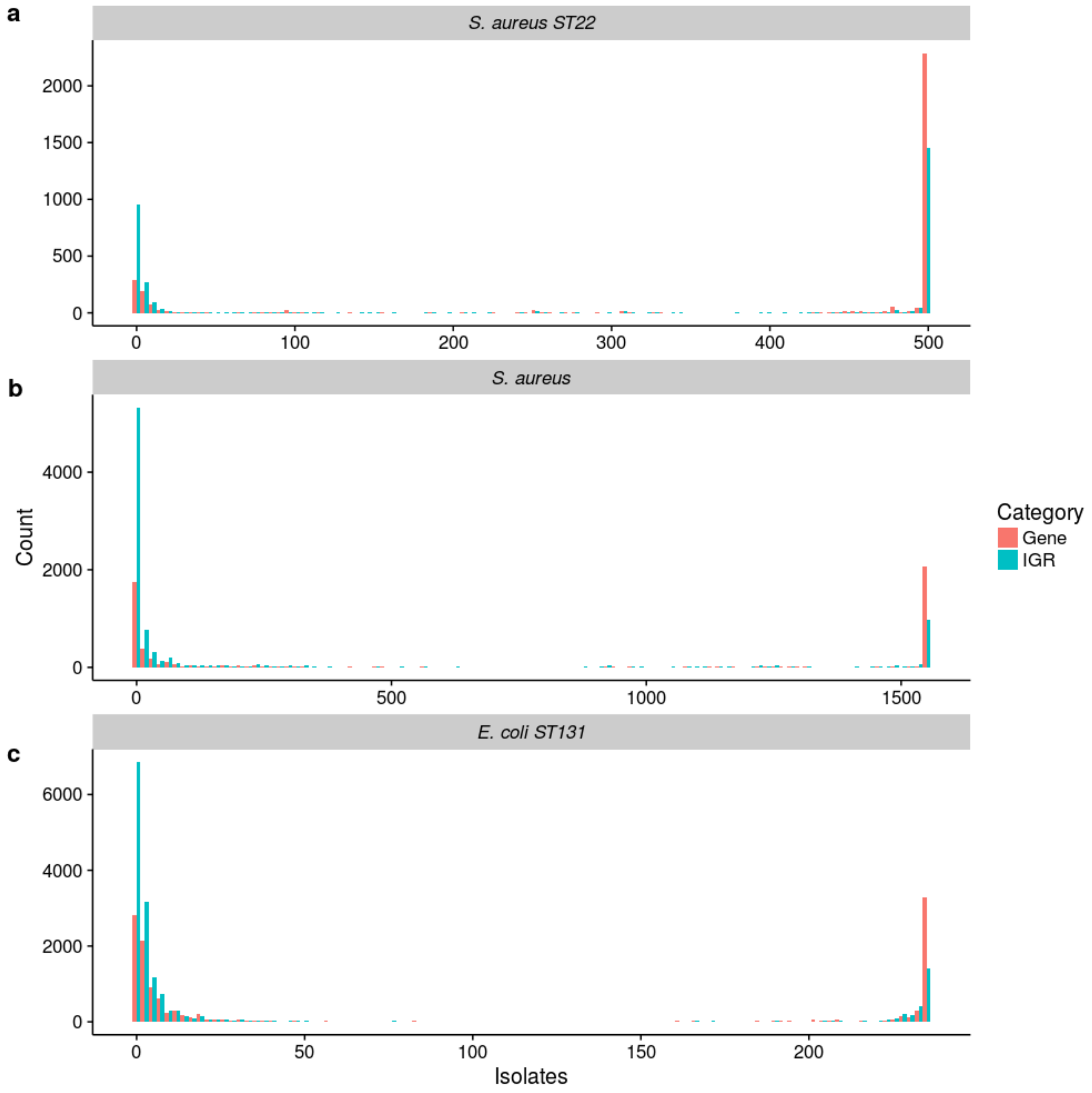

Fig 2: Properties of the pan-genomes. Genes (red) and IGRs (blue) were analysed with frequency histograms (the number of genes/IGRs present in any given number of isolates). The vast majority of genes / IGRs are either very rare or very common. a) *S. aureus* ST22 b) *S. aureus* c) *E. coli* ST131.

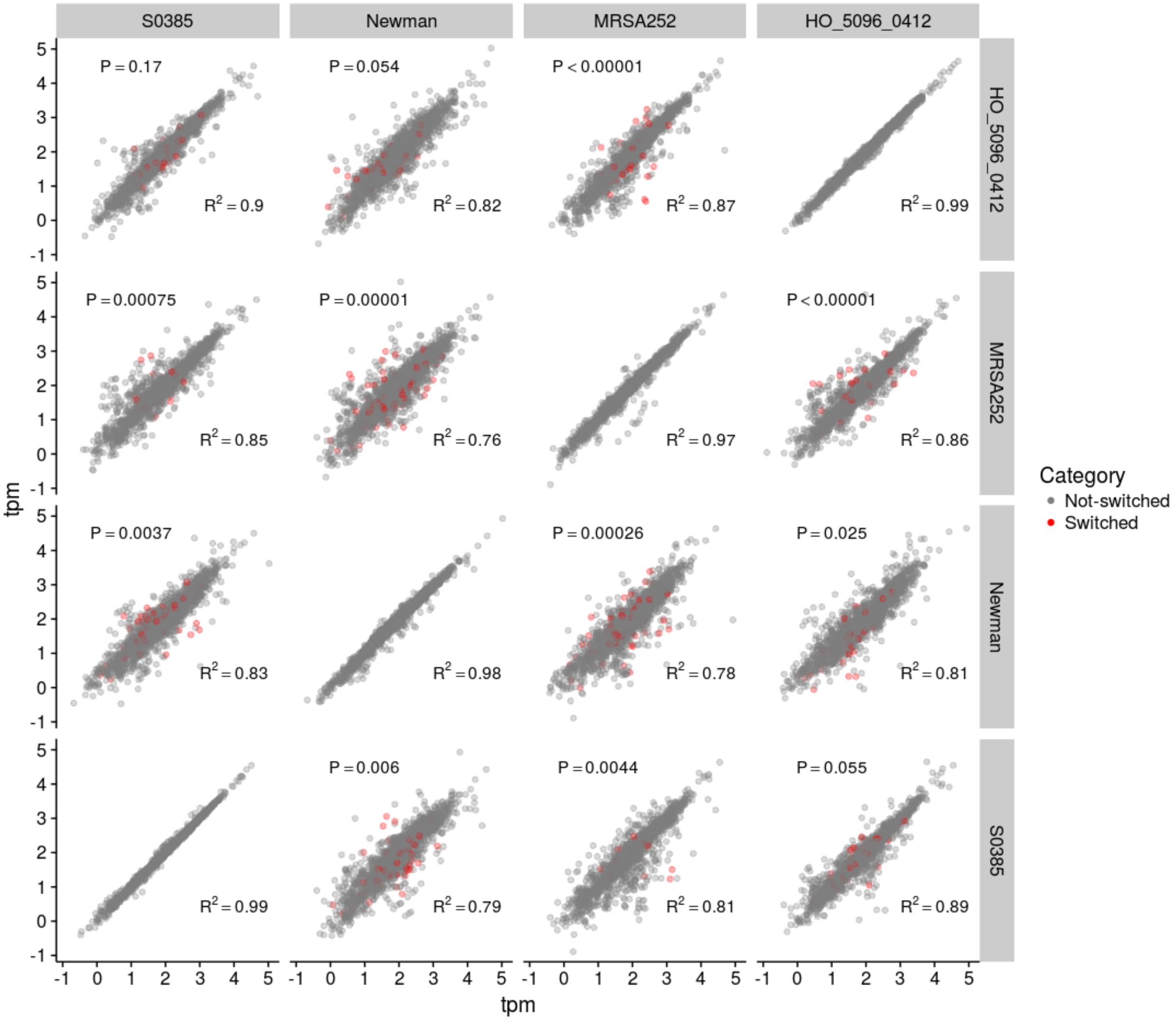

Fig 3: *S. aureus* gene expression data. Pairwise RNA-seq comparisons between four *S. aureus* isolates, where two biological replicates were used for each isolate. The top-left of the diagonal corresponds to comparisons between replicate 1 from different isolates (e.g. SO385 replicate 1 vs HO_5096_0412 replicate 1). The bottom-right of the diagonal corresponds to comparisons between replicate 2 from different isolates (e.g. SO385 replicate 2 vs HO_5096_0412 replicate 2). The diagonal corresponds to comparisons between the two biological replicates from the same isolate. 2094 core genes were analysed in each comparison,

and tpm (Transcripts per Kilobase Million) was used to quantify expression. The genes were separated into two categories: Switched (red), and Not-switched (grey), based on their upstream IGRs. The R^2 value corresponds to all the genes. The P-value corresponds to a Monte Carlo permutation test comparing the residuals of the two groups of genes, where a significant score indicates that the genes downstream of switch IGRs are associated with a higher degree of differential expression (ie higher residuals).

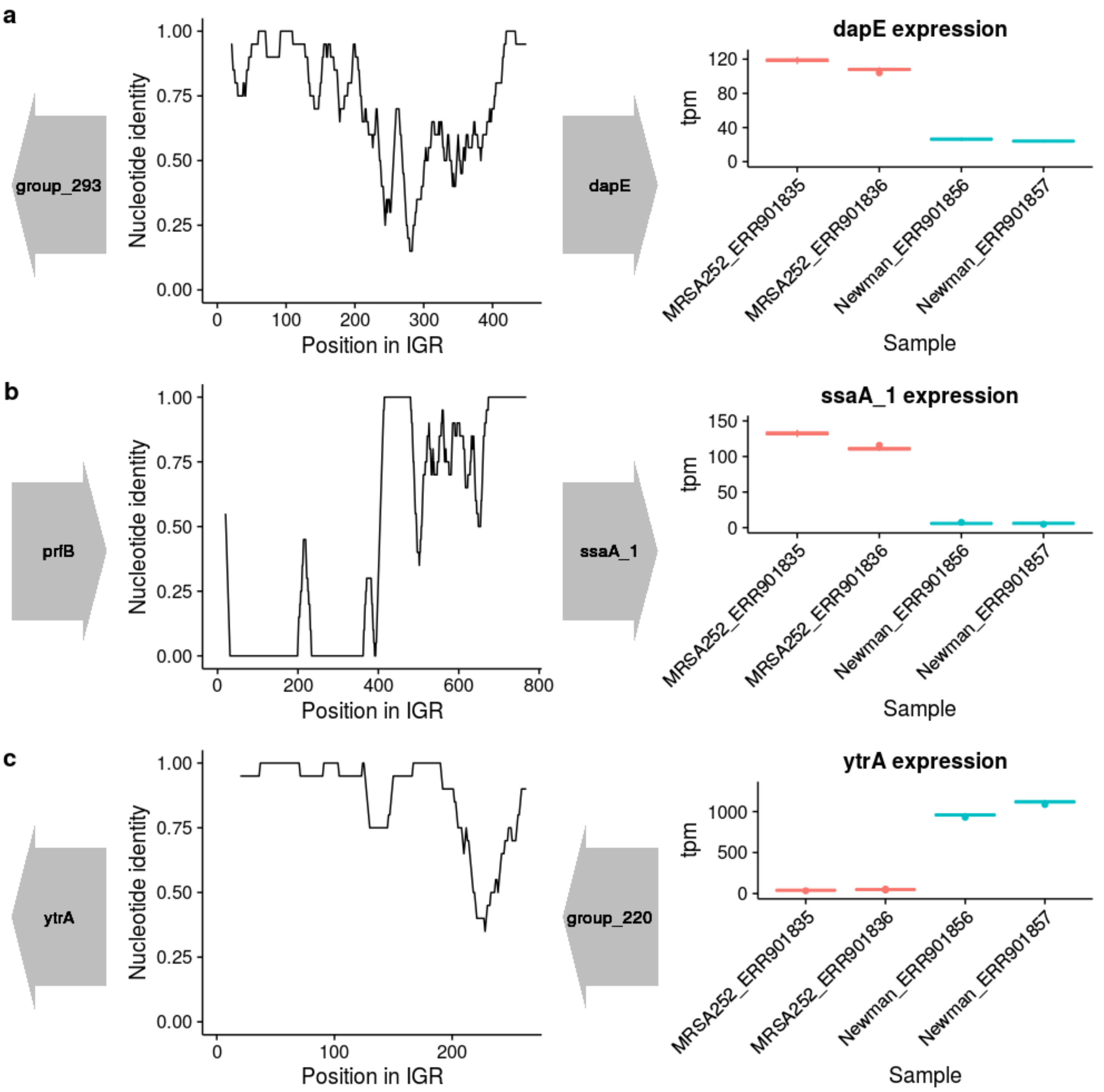

Fig 4: A detailed view of the genomic neighbourhood and expression data for selected genes in Newman vs MRSA252. Nucleotide identity was calculated using a 20 bp sliding window across the IGR, and this is shown alongside the flanking genes in their correct orientation (left). The corresponding expression data for the gene of interest was also shown (right), with the two boxplots per isolate corresponding to the two biological replicates. a) dapE b) ssaA_1 c) ytrA.

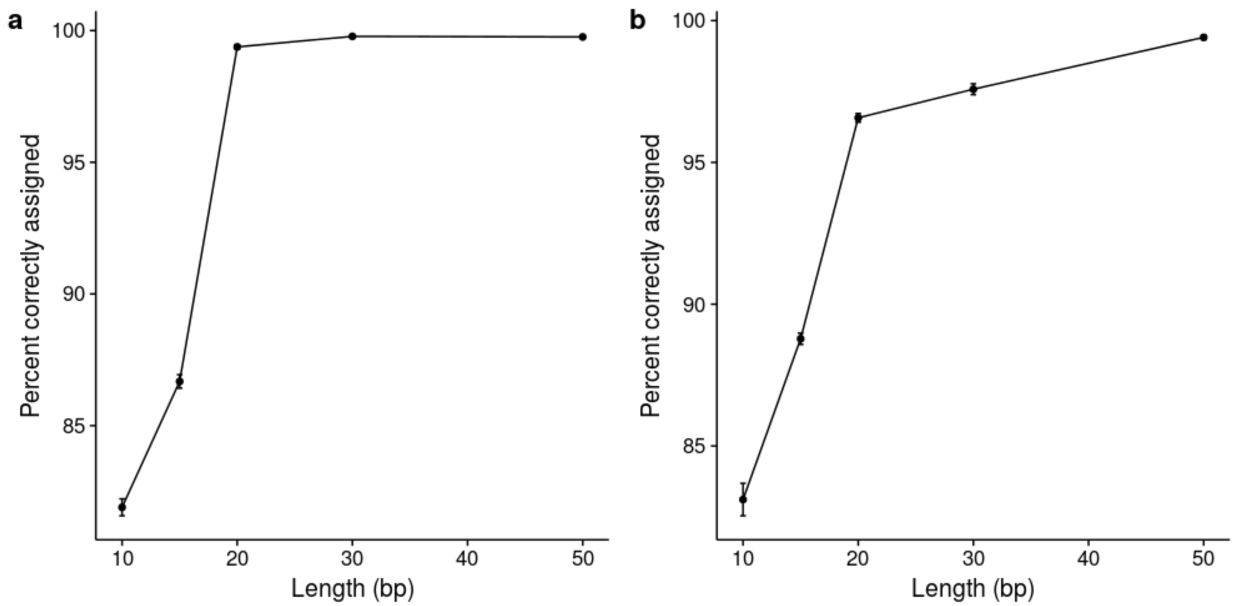

Fig S1: Clustering performance. The clustering performance was assessed by truncating IGR sequences and reclustering them with the pool of original sequences. Truncated IGR which were placed into the same cluster as their progenitor sequences were deemed to be correctly clustered. a) *S. aureus* b) *E. coli*.

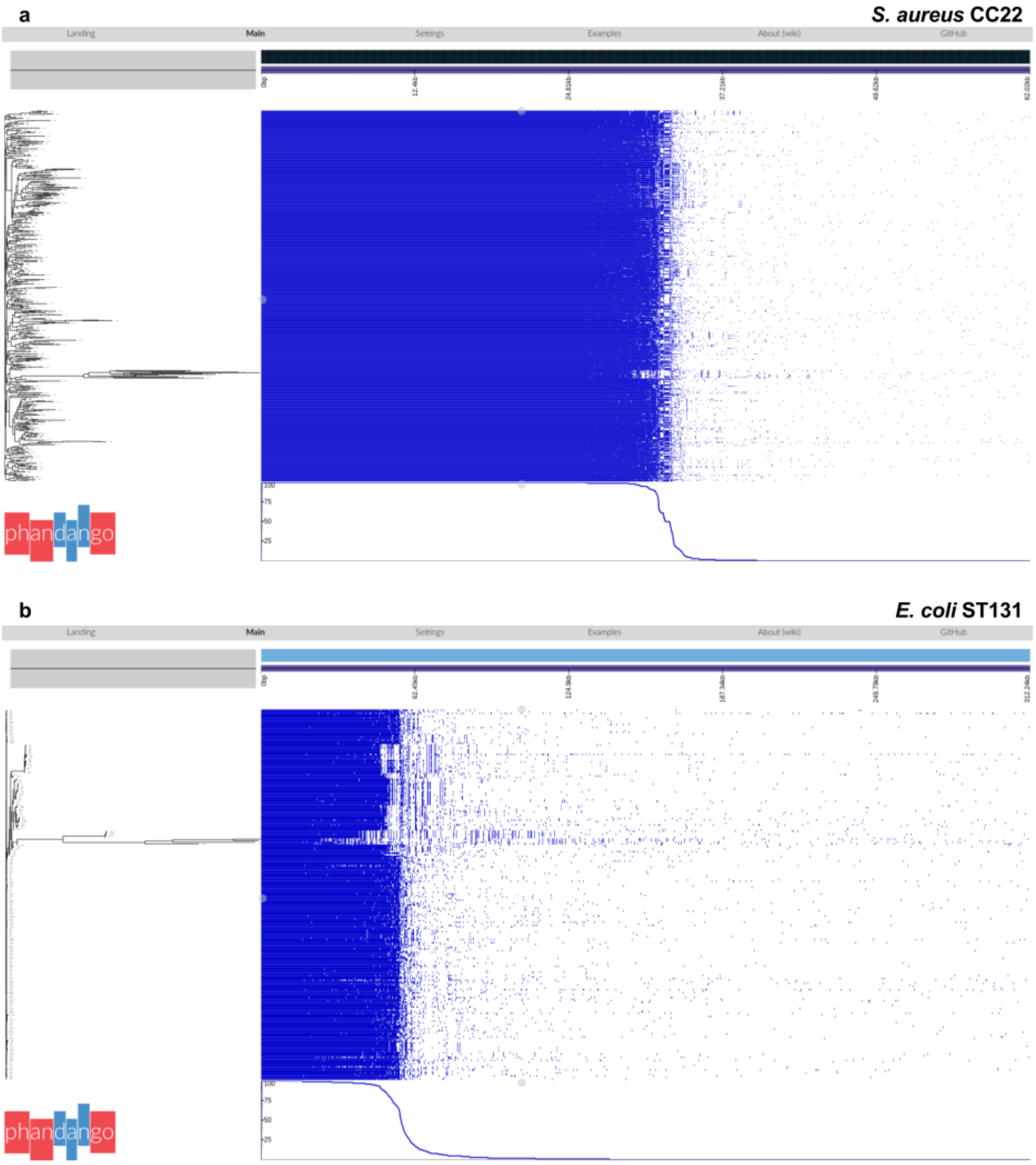

Fig S2: The IGR pan-genome ("panIGRome") as visualised using Phandango. A neighbour-joining phylogenetic tree was imported into Phandango alongside the IGR_presence_absence.csv file. Each row corresponds to an isolate, and each column corresponds to an IGR, with the IGRs ordered from the left in order of decreasing frequency

within the sample. The line graph at the bottom shows the frequency of the IGRs within the sample. a) *S. aureus* ST22 b) *E. coli* ST131.

Sheet1

**Species**	**Core genes**	**Core IGRs**	**Accessory genes**	**Accessory IGRs**	**Percentage core genes**	**Percentage core IGRs**
S. aureus ST22	2409	1556	816	1543	95	95
S. aureus	2129	1134	3446	8033	85	69
E. coli ST131	3930	2296	8876	14133	84	77

 Click here to download Table Table_2.xlsx

Sheet1

**Species**	**Core gene, Core IGR**	**Core gene, Accessory IGR**	**Accessory gene, Core IGR**
S. aureus ST22	99.5	0.5	7.4
S. aureus	92.9	7.1	3.2
E. coli ST131	97.9	2.1	2.7

Accessory gene, Accessory IGR
92.6
96.8
97.3

Click here to access/download
Supplementary Material
Table_S1 (1).xlsx
