# Supplementary material for: Piggy: a rapid, large-scale pan-genome analysis tool for intergenic regions in bacteria
Source: Gigascience. 2018 Mar 4;7(4):giy015. doi: 10.1093/gigascience/giy015 (PMC5890482; doi:10.1093/gigascience/giy015)
Supplement: GIGA-D-17-00244_Revision_2.pdf [file giy015_giga-d-17-00244_revision_2.pdf]

GigaScience

Piggy: A Rapid, Large-Scale Pan-Genome Analysis Tool for Intergenic Regions in Bacteria

--Manuscript Draft--

**Manuscript Number:**	GIGA-D-17-00244R2
**Full Title:**	Piggy: A Rapid, Large-Scale Pan-Genome Analysis Tool for Intergenic Regions in Bacteria
**Article Type:**	Technical Note
**Funding Information:**	Medical Research Council (G1000803) — Not applicable<br>United Kingdom Clinical Research Collaboration Translational Infection Research Initiative — Not applicable
**Abstract:**	Abstract<br>Background<br>The concept of the "pan-genome", which refers to the total complement of genes within a given sample or species, is well established in bacterial genomics. Rapid and scalable pipelines are available for managing and interpreting pan-genomes from large batches of annotated assemblies. However, despite overwhelming evidence that variation in intergenic regions in bacteria can directly influence phenotypes, most current approaches for analysing pan-genomes focus exclusively on protein-coding sequences.<br>Findings<br>To address this we present Piggy, a novel pipeline that emulates Roary except that it is based only on intergenic regions. A key utility provided by Piggy is the detection of highly divergent ("switched") IGRs upstream of genes. We demonstrate the use of Piggy on large datasets of clinically important lineages of Staphylococcus aureus and Escherichia coli.<br>Conclusions<br>For S. aureus, we show that highly divergent ("switched") IGRs are associated with differences in gene expression, and we establish a multi-locus reference database of IGR alleles (igMLST; implemented in BIGSdb). Piggy is available at https://github.com/harry-thorpe/piggy.
**Corresponding Author:**	Edward Feil<br><br>UNITED KINGDOM
**Corresponding Author Secondary Information:**	
**Corresponding Author's Institution:**	
**Corresponding Author's Secondary Institution:**	
**First Author:**	Harry A. Thorpe
**First Author Secondary Information:**	
**Order of Authors:**	Harry A. Thorpe<br>Sion C. Bayliss<br>Samuel K. Sheppard<br>Edward J. Feil
**Order of Authors Secondary Information:**	
**Response to Reviewers:**	To the editor:<br><br>Thank you for accepting our manuscript for publication in GigaScience.

Powered by Editorial Manager® and ProduXion Manager® from Aries Systems Corporation

	We do not feel that it is necessary to host the supporting data in GigaDB as all the data is already published and available at public repositories as described in the manuscript.<br><br>Although in principle Code Ocean looks to be valuable, we do not think it is appropriate for Piggy for a number of reasons. First, Piggy requires various dependencies (particularly Roary), which are not installed on Code Ocean. Second, the free plan only provides 5 GB of storage and 1 compute hour per month - these are not sufficient for any real analysis of whole-genome sequence data. We will however consider it in any future projects.<br><br>We have reformatted the manuscript as per the guidelines.<br><br>To the reviewer:<br><br>We thank the reviewer for their detailed, constructive review, which helped us to improve the manuscript.
**Additional Information:**	
**Question**	**Response**
Are you submitting this manuscript to a special series or article collection?	No
**Experimental design and statistics**<br><br>Full details of the experimental design and statistical methods used should be given in the Methods section, as detailed in our Minimum Standards Reporting Checklist. Information essential to interpreting the data presented should be made available in the figure legends.<br><br>Have you included all the information requested in your manuscript?	Yes
**Resources**<br><br>A description of all resources used, including antibodies, cell lines, animals and software tools, with enough information to allow them to be uniquely identified, should be included in the Methods section. Authors are strongly encouraged to cite Research Resource Identifiers (RRIDs) for antibodies, model organisms and tools, where possible.<br><br>Have you included the information requested as detailed in our Minimum Standards Reporting Checklist?	Yes
**Availability of data and materials**<br><br>All datasets and code on which the conclusions of the paper rely must be	Yes

either included in your submission or deposited in publicly available repositories (where available and ethically appropriate), referencing such data using a unique identifier in the references and in the "Availability of Data and Materials" section of your manuscript.<br><br>Have you have met the above requirement as detailed in our Minimum Standards Reporting Checklist?	

Manuscript Click here to download Manuscript Piggy16-2.docx

Piggy: A Rapid, Large-Scale Pan-Genome Analysis Tool for Intergenic Regions in Bacteria.

Harry A. Thorpe[1], Sion C. Bayliss[1], Samuel K. Sheppard[1], Edward J. Feil[1*]

[1]The Milner Centre for Evolution, Department of Biology and Biochemistry, University of Bath, Bath BA2 7AY.

*Corresponding author

h.thorpe@bath.ac.uk

s.bayliss@bath.ac.uk

s.k.sheppard@bath.ac.uk

e.feil@bath.ac.uk

Keywords: Pan-genome, Accessory genome, Genomics, Whole-genome sequencing (WGS), Bacteria, Intergenic Regions, igMLST, Gene Expression, *Staphylococcus aureus*, *Escherichia coli*.

RRID: (Piggy, RRID:SCR_015941)

Abstract

Background

The concept of the “pan-genome”, which refers to the total complement of genes within a given sample or species, is well established in bacterial genomics. Rapid and scalable pipelines are available for managing and interpreting pan-genomes from large batches of annotated assemblies. However, despite overwhelming evidence that variation in intergenic regions in bacteria can directly influence phenotypes, most current approaches for analysing pan-genomes focus exclusively on protein-coding sequences.

Findings

To address this we present Piggy, a novel pipeline that emulates Roary except that it is based only on intergenic regions. A key utility provided by Piggy is the detection of highly divergent (“switched”) IGRs upstream of genes. We demonstrate the use of Piggy on large datasets of clinically important lineages of *Staphylococcus aureus* and *Escherichia coli*.

Conclusions

For *S. aureus,* we show that highly divergent (“switched”) IGRs are associated with differences in gene expression, and we establish a multi-locus reference database of IGR alleles (igMLST; implemented in BIGSdb). Piggy is available at https://github.com/harry-thorpe/piggy.

Findings

Introduction

Whole-genome sequencing has revealed that, in many bacteria, individual strains frequently recruit new genes from a seemingly endless genetic reservoir [1,2]. The total complement of genes observed across all strains, known as the pan-genome, often numbers tens of thousands, up to an order of magnitude more than the number of genes present in any single genome. In contrast, the “core-genome”, which refers to the complement of genes present in all (or the vast majority) of sampled isolates, can be significantly smaller than the total number of genes in any given genome [3,4]. For example, a study of 328 *Klebsiella pneumoniae* isolates, each of which harbour 4-5,000 genes, revealed a pan-genome of 29,886 genes; only 1,888 (6.8%) of which were universally present (core) [5]. Similarly, genome data for 228 *Escherichia coli* ST131 isolates revealed a pan-genome of 11,401 genes, of which 2,722 (23.9%) were core [6]. The degree of gene content variation in the latter study is particularly striking as these isolates were all from the same sequence type (ST), thus show limited nucleotide divergence in core genes, and are descended from a recent common ancestor. More generally, the relationship between the size of the core and accessory genomes varies between species, with

ecologically diverse species having large accessory genomes, and ecologically restricted species (such as endosymbionts) having small accessory genomes [1,2].

There is growing recognition that the acquisition of new genes through horizontal gene transfer (HGT) has a central role in ecological adaptation [7]. The emergence and spread of antibiotic resistance, underpinned by the transfer of plasmids and other mobile genetic elements (MGEs), is a pertinent example. The increasing availability of datasets containing thousands of isolates thus offers an unprecedented opportunity for describing the genetic basis of bacterial adaptation, although the scale of these data presents serious logistic and conceptual challenges in terms of data management and analysis.

Pioneering pan-genome analysis tools, such as PanOCT and PGAP relied on all-vs-all BLAST comparisons between protein sequences, and scaled approximately quadratically with the number of isolates [8,9]. LS-BSR introduced a pre-clustering step which substantially reduced the number of BLAST comparisons, enabling it to be feasibly run on thousands of samples [10]. More recently, the Roary pipeline has rapidly gained popularity for scalable, user-friendly, pan-genome characterisation [4].

The concept of the pan-genome, as described above, places an exclusive emphasis on genes; or, more specifically, open reading frames with the potential to encode proteins. This gene-centric perspective has both shaped, and been shaped by, the bioinformatics tools developed to interrogate the pan-genome. For example, Roary works by taking individual protein-coding sequences, pre-defined using Prokka annotation [11], and assigning each to a single cluster of homologous sequences. This approach thus excludes non protein-coding intergenic regions (IGRs) which typically account for approximately 15% of the genome [12,13]. This is clearly problematic for downstream attempts to identify genotype-phenotype links, as IGRs contain many important regulatory elements including, but not limited to, promoters, terminators, non-coding RNAs, and regulatory binding sites. Moreover, we have recently shown that IGRs are subject to purifying selection in the core-genomes of diverse bacterial species, even when known major regulatory elements are excluded [14,15], and a recent study has shown that intergenic variation is positively selected during *Pseudomonas aeruginosa* infections [16].

Given that variation in IGRs can have profound phenotypic consequences, it is timely to consider how best to incorporate these sequences into pan-genome analyses. A key question is

the degree to which protein-coding genes, and their cognate regulatory elements, should be considered a single "unit", both selectively (in terms of co-adaptation) and in terms of physical linkage on the chromosome. If physical linkage is assumed to be highly robust, such that genes are mostly transferred along with their cognate IGRs, then in principle the definition of a "gene" could be expanded to include the upstream regulatory regions. On the other hand, if there is moderate or weak linkage between genes and IGRs, such that IGRs can occasionally transfer independently, then the purview of the pan-genome could be expanded to include the full complement of IGR alleles in addition to protein-coding sequences.

Consistent with the second model, which allows for independent transfer of IGRs, a landmark study demonstrated that *E. coli* genes can apparently be regulated by alternative IGRs that frequently share no sequence similarity to each other [17]. Moreover, the distribution of these IGRs was incongruent with gene trees, suggesting that recombination can act to replace one IGR with another resulting in regulatory "switches"; a process they call horizontal regulatory transfer (HRT) [17]. It is important to note here that the term "switching" refers only to the replacement of an IGR by a non-homologous or highly divergent variant sequence. It does not specify that the replacement IGR has a particular origin, and could therefore correspond to a transfer from elsewhere in the same genome, or from another isolate. It was also noted that conserved flanking genes may facilitate this process by providing localised regions of homology. IGR switches can be accompanied by differential gene expression [17], and may provide a mechanism to offset the fitness costs of harbouring plasmids and other MGEs [6], pointing to a central role for this process in adaptation.

Our current understanding of the evolutionary dynamics of IGRs in the context of bacterial pan-genomes leaves many open questions. Specifically, it is unclear how IGRs are distributed among isolates within bacterial populations, how commonly IGRs and their cognate genes are co-transferred, or how the frequency of HRT relates to different functional gene categories. A more complete understanding of bacterial adaptation clearly requires a careful consideration of gene presence/absence alongside gene regulation. Here we address this by introducing a new pipeline called Piggy which closely emulates and complements the established pan-genome analysis pipeline Roary [4]. Input and output files for Piggy and Roary use the same format, and run in a similar time on modest computing resources. Piggy provides a means to rapidly identify IGR switches, and more broadly the means to examine the role of horizontal transfer in shaping the bacterial regulome. We demonstrate the utility of Piggy using large genome datasets for

two bacterial species, both of which are of high public health importance; *Staphylococcus aureus* and *Escherichia coli*. Conventional pan-genome analyses are applied to analyse and compare core and accessory IGRs/genes in these lineages. In *S. aureus* we show an association between IGR switching and changes in gene expression, and demonstrate proof-of-principle by establishing a multilocus IGR scheme, (igMLST) in BIGSdb [18]. Piggy is available at (https://github.com/harry-thorpe/piggy) under the GPLv3 licence.

Methods

Datasets

The *S. aureus* dataset was assembled from published genome sequences [19] available from the European Nucleotide Archive (ENA), study number ERP001012. The *S. aureus* RNA-seq data was previously published [20], and is available from the ENA, study number ERP009279. This was supplemented with the corresponding reference genomes, HO_5096_0412: HE681097, MRSA252: BX571856, Newman: AP009351, S0385: AM990992, available from the National Center for Biotechnology Information (NCBI). The *E. coli* ST131 dataset was from a previously published study [6], and is available at [21]. All complete genomes and assemblies were annotated with Prokka [11].

Roary and Piggy parameter settings

Roary [4] was run using default parameters except for the following: -e -n (to produce alignments with MAFFT [22]); -i 90 (lower amino acid identity than the default); -s (to keep paralogs together); -z (to keep intermediate files). Piggy was run using default parameters except for --len_id, which controls the percentage of IGR sequences which must share similarity in order to be clustered together. For the *S. aureus* and *E. coli* ST131 datasets, Piggy was run twice, once with --len_id 10 and once with --len_id 90. The former was used for the pan-genome comparisons between genes and IGRs (Fig 2) in order to be comparable with Roary. Using a low length identity (--len_id 10) enabled homologous sequences of varying lengths (for example a truncated sequence) to cluster together. Roary does not provide a similar setting, and only requires that sequences have a minimum length of 120 bp. Genes in the same clusters defined by Roary may vary considerably in length, either due to genuine truncations or assembly errors. A relaxed --len_id setting of 10 was therefore used in Piggy to provide consistency with Roary and to ensure that homologous IGRs are not erroneously placed in different clusters. A --len_id setting of 90 was subsequently used whenever “switched” IGRs were detected, as this enabled sequences to be subsequently filtered by either nucleotide or length identity.

RNA-seq analysis

Two biological replicates for each isolate were analysed. Kallisto [23] was used to quantify transcripts (--kmer-size 31 and --bootstrap-samples 100), and Sleuth [24] was used to normalise and filter the counts produced by Kallisto. These counts were then $\log_{10}$ transformed, and major axis (MA) regression was performed. Rockhopper2 [25] was used to produce an operon map for each strain by grouping adjacent genes with similar expression profiles together into operons.

Clustering performance

We examined the clustering performance of Piggy by producing truncated variants of IGRs of lengths 10,15,20,30,50 bp, and comparing how the lengths of the IGRs altered the resulting clustering. The IGRs were truncated from a random starting point in the sequence, and each length was analysed separately. From the starting pool of IGRs from 10 randomly selected isolates, 1000 IGRs were chosen and truncated. These truncated variants were then added to the pool of IGRs and Piggy was run on them. Clustering patterns based on the truncated and original IGRs were then compared, with truncated IGRs placed in the same cluster as their progenitor sequences being assigned as correctly clustered. This analysis was performed on both the *S. aureus* ST22 and *E. coli* ST131 datasets.

Statistical analysis

All statistical analysis was performed within R version 3.3.2 [26]. All plotting was performed with ggplot2 [27].

Results

Overview of the Piggy pipeline

Fig 1a shows an overview of the Piggy pipeline. The first step is to run Roary, as the gene presence absence output file from Roary is used as an input for Piggy. Piggy is then run using the same annotated assemblies as Roary, specifically GFF3 format files such as those produced by Prokka [11]. Piggy extracts intergenic sequences (IGRs) from these files, and uses the flanking gene names and their orientations to name the IGRs (Fig 1b). Each IGR name contains three pieces of information: the upstream gene, the downstream gene, and their relative orientations (CO - Co-Oriented, DP - Double Promoter, DT - Double Terminator). For example, the IGR “Gene_1 Gene_2 DP” is flanked by Gene_1 and Gene_2, which are both downstream of the IGR (i.e. they are transcribed in opposite directions). IGRs at the edge of

contigs are excluded by default, but when they are included (using the --edges flag) the missing information is denoted by NA, for example “Gene_1 NA NA”. Including the gene neighbourhood information gives context to the IGR and enables identification of “switched” IGRs. By default, only IGRs between 30-1000 bp in length are included by Piggy, though these lengths can be user-defined using the --size flag (minimum length = 30 bp). The IGRs are then clustered with CD-HIT [28] at user-defined identity thresholds (--nuc_id - nucleotide identity, --len_id - length identity). The nucleotide identity is defined as SNPs / aligned sites, and the length identity is defined as shared sites / alignment length. These two flags allow the user to set the level of stringency for clustering. For example, a conservative approach is to set high values for both nucleotide and length identity such that IGRs must be similar in both nucleotide and length identity to cluster together. By relaxing the length identify whilst maintaining a high nucleotide identity threshold, highly related sequences still cluster even if one is truncated. The longest sequence from each cluster is then used to perform an all-vs-all BLASTN search [29]. This is used to merge similar clusters (BLASTN defaults, except -word_size = 10), which did not cluster with CD-HIT. These clusters are then used to produce an IGR presence absence matrix (“IGR_presence_absence.csv”), in the same format as the gene presence absence matrix (“gene_presence_absence.csv”) produced by Roary. Up until this point, the pipeline is very similar to Roary [4].

Switched IGR detection

Piggy identifies “switched” IGRs using two methods. For both methods, the term “switch” refers to two or more divergent IGR sequences occupy the same locus as defined by flanking genes, but does not specify an origin for the divergent IGR sequences [17]. The first method identifies adjacent genes on the same contig (gene-pairs), and searches for IGR clusters which lie between these gene-pairs (Fig 1c). Instances where multiple IGR clusters correspond to the same gene-pair are identified as candidate switched IGRs. The second method identifies instances where multiple IGR clusters occupy a locus upstream of a single gene cluster. This is a less conservative approach as only one of the two genes flanking the IGR is taken into account, (Fig 1c). The gene-pair method is used by default as it controls against detecting “switching” (recombination) events that encompass more than a single IGR, for example, cases where a mobile element has inserted between two genes. However such cases remain relevant as the regulation of the downstream gene may still be affected.

To ensure that differences in gene annotation between isolates, specifically artefactual variation in the start and end points of each gene, are not erroneously assigned as switching events, the first and last 30 bp of each flanking gene are searched against the IGRs with BLASTN. Any matches from these searches indicate differences in annotation of gene borders (rather than genuine differences between the IGRs), and these sequences are disregarded. In order to confirm that they represent genuine switching events, candidate switched IGRs are searched against each other with BLASTN with low complexity filtering turned off (-dust no). If there is no significant match they are classed as “switched”, and if there is a significant match they are aligned using MAFFT [22]. The resulting alignment is then used to calculate nucleotide identity (SNPs / shared sites), and length identity (number of shared sites / alignment length). These values can then be used to define an appropriate threshold to identify “switched” IGRs. To aid this, Piggy calculates within-cluster divergences for both genes and IGRs, and these divergences can be used to calibrate Piggy with Roary.

Clustering performance

The shorter lengths of IGRs compared with genes poses potential problems for alignment accuracy. We tested the clustering performance of Piggy by producing truncated variants of IGRs, adding these to the total complement of IGRs in an analysis, and then recording whether the truncated IGRs were clustered with their untruncated counterparts (Methods). For *S. aureus* ST22, 82% of IGRs truncated to 10 bp clustered together with the corresponding full length sequences, but this figure increased to > 99% when the length of the truncated sequences was 20 bp. (Fig S1a). A similar increase was observed for the *E. coli* ST131 data, although in this case 50 bp was required for the percentage of correct assignments to be > 99%. (Fig S1b).

An inspection of the incorrectly clustered sequences from both datasets revealed that their progenitor sequences shared high sequence similarity in parts of their sequence to other IGR clusters, but no sequence similarity in other parts of the sequence. This resulted in separate clusters which shared high sequence homology over parts of their sequences. When these sequences were truncated to assess the clustering, if the homologous part of the sequence was selected, then it could align to either of these progenitor IGR clusters. In many cases these alignments were perfect matches, and so the IGR could not be unambiguously placed. This problem is likely to be a result of non-homologous breaks at the edge of HGT events, and this is consistent with greater clustering accuracy in *S. aureus* ST22 compared with *E. coli* ST131, where the latter has a much larger pan-genome.

Staphylococcus aureus

S. aureus is an important skin-associated bacterium which is commonly carried asymptomatically, but can also cause a wide range of infections from minor skin infections to fatal bacteraemias. It has a clonal population structure consisting of discrete lineages [30]. Although the core genome is relatively stable, phenotypic variation (e.g. resistance profiles, virulence traits, and host preference) is associated with a more dynamic accessory genome and the horizontal transfer of MGEs, such as the SCC*mec* element which confers resistance to β-lactam antibiotics [31].

S. aureus ST22 (EMRSA-15) is a clinically important hospital-acquired methicillin resistant strain which is common in the UK and is rapidly expanding elsewhere in Europe and globally [32]. Previous work has shown that *S. aureus* ST22 is clonal and shows relatively little variation in gene content [19,32]. In order to compare the pan-genomes of *S. aureus* at different scales, we analysed a diverse dataset of 1552 isolates from many lineages, and a smaller dataset of 500 ST22 isolates subsampled from the larger dataset [19]. The size of the gene and IGR pan and core-genomes were compared by analysing both datasets with Roary and Piggy. Frequency histograms were plotted for both genes and IGRs (Fig 2a-b).

The gene-IGR frequency histogram for ST22 (Fig 2a) shows that there are 2,409 core genes and 1,556 core IGRs, where core is defined as gene presence in > 95% of isolates (Table 1). When the whole species is considered, these numbers drop to 2,129 and 1,134, respectively. The fact that there are fewer core IGRs than core genes is in part due to the exclusion of IGRs < 30 bp (many of which are intra-operonic), but also likely reflects faster evolution of IGRs. Both distributions conform to the U-shape typically found in such analyses where the majority of genes/IGRs are either very common or very rare, however the distribution of genes and IGRs is shifted towards the rare sequences when the whole species is considered rather than only ST22.

We used the output of Piggy to investigate the degree of linkage between genes and IGRs. We identified all genomic loci consisting of an IGR flanked by two genes, and from these we identified all pairs of genes and IGRs where the IGR was upstream of the gene start. We then grouped these according to whether the gene or IGR was core or accessory (Table 2). For the *S. aureus* ST22 data, 99.5% of core genes were immediately downstream of a core IGR, and

92.9% of the accessory genes were similarly downstream of an accessory IGR. When considering the wider *S. aureus* dataset the figures were similar; 92.6% of core genes were downstream of a core IGR, and 96.8% of accessory genes were downstream of an accessory IGR. Thus, the assignment of an IGR as core or accessory is strongly predictive of the corresponding assignment of the cognate downstream gene, which in turn points to strong background linkage between genes in IGRs in the genome.

Escherichia coli ST131

The utility of Piggy was further validated by re-analysing data from a recent study on the widespread and clinically important *E. coli* lineage ST131 [6]. This dataset contains 228 clinical *E. coli* ST131 isolates from human, domesticated animal, and avian hosts. *E. coli* is a more genetically diverse species than *S. aureus*, and unsurprisingly *E. coli* ST131 has a larger pan-genome than *S. aureus* ST22, with 12,806 genes and 16,429 IGRs (Fig 2c, Table 1). More surprisingly, *E. coli* ST131 has a larger pan-genome than the whole *S. aureus* species. Within *E. coli* ST131, 3,930 genes and 2,296 IGRs were core out of an average of 4,689 genes and 2,984 IGRs per isolate. Thus despite the differences between the two species in their level of diversity there was a consistent signal of a lower number of core IGRs than core genes, and a high number of accessory IGRs compared to accessory genes. There was tight linkage between genes and IGRs, with 97.9% of core genes being immediately downstream of core IGRs and 97.3% of accessory genes being similarly downstream of accessory IGRs; these results are consistent with those from *S. aureus* (Table 2).

The data from *S. aureus* and *E. coli* shows a background of strong linkage between core genes and IGRs. However, this linkage is not perfect; some core genes are associated with accessory IGRs (and vice-versa), and the linkage is weaker over long timescales (across the whole *S. aureus* species compared to within ST22). Previous work has examined this linkage and found evidence of widespread IGR regulatory switching, where genes are regulated by alternative IGRs in different isolates [17]. Piggy provides a list of candidate switching events together for both “gene-pair” and “upstream” approaches (see Methods) at different thresholds of nucleotide identity. For the *E. coli* ST131 data, the pipeline detected 61 cases of putative IGR switching using the most conservative settings (i.e. the conservative gene-pair method, and the alternative IGRs showing no sequence similarity by BLASTN). Relaxing the threshold of sequence identity to < 90% resulted in the identification of an additional 317 candidate switching events, though these possibly reflect either relaxed or positive selection.

Switched IGRs influence gene expression in *S. aureus*

To examine whether switches in IGRs affect the expression of cognate (downstream) genes, we used a previously published RNA-seq dataset based on four reference *S. aureus* isolates HO_5096_0412 (ST22), Newman (CC8), MRSA252 (CC36), and S0385 (CC398) [20]. Each of these *S. aureus* references isolate represents a distinct major clonal complex, and all were grown under identical conditions with each experiment being replicated. Thus these data provide evidence of the natural variation in gene expression within the *S. aureus* population. By analysing these data alongside the output from Piggy, it is possible to test the extent to which IGR switches between these four genomes can account for the observed variation in gene expression between clonal complexes. First Roary was used to identify a set of 2094 single copy core genes present in all four isolates, and then expression of these core genes was quantified using Kallisto [23]. To do this we used RNA-seq data for two replicates for each of the four reference genomes. The tpm (Transcripts per Kilobase Million) values for each gene are given in Table S1. We then used Sleuth [24] to normalise and filter these counts.

To check the consistency of the data between biological replicates, we first plotted two replicates for each isolate against each other (e.g. Newman replicate 1 vs Newman replicate 2) (Fig 3). These plots were tightly correlated (mean R^2 = 0.98), confirming that the expression values for individual genes were consistent between replicates. We then plotted between-isolate comparisons, again using both replicates for each genome (e.g. Newman replicate 1 vs MRSA252 replicate 1, and Newman replicate 2 vs MRSA252 replicate 2) (Fig 3). These comparisons revealed considerably more scatter, with R^2 values ranging from 0.76 to 0.9. Given the extremely high R^2 values for within-isolate comparisons, the decrease in R^2 for between-isolate comparisons reflects genuine differences in expression between the isolates. We note that a small number of genes show very striking differences in expression between the clonal complexes. For example, the expression of *mepA*, which encodes a multidrug efflux pump, was ~250 fold higher in Newman compared with the other isolates.

The genomes of each pair of isolates were analysed using Roary and Piggy to identify switched IGRs with a nucleotide identity threshold of < 90% for IGR clusters. For each pair of isolates, we then identified all genes immediately downstream of a switched IGR. As a single switched IGR might impact on the expression of more than one co-transcribed downstream genes we also considered all genes linked in a single operon that could be impacted by a single switching

event upstream affecting a shared promoter. For each pair of isolates, we thus identified all core genes putatively affected by upstream IGR switches. We then tested whether these genes showed a higher degree of differential expression by conducting Monte Carlo permutation tests on the residuals from the regressions (Fig 3). For each pairwise comparison of isolates, we summed the residuals of the genes with switched IGRs (shown as red points in Fig 3), and compared this to a distribution obtained by resampling (without replacement) 100,000 random sets of the same number of genes and summing their residuals. We computed a one-tailed p-value by dividing the number of permutations with summed residuals greater than the observed value by 100,000 (Fig 3). Because we used both replicates separately (e.g. Newman replicate 1 vs S0385 replicate 1, and Newman replicate 2 vs S0385 replicate 2), each comparison between pairs of isolates was tested twice. In 9/12 pairwise comparisons, the observed residuals of the genes downstream of switched IGRs were significantly ($p < 0.05$) greater than expected from the resampled data, indicating that genes with switched IGRs were more differentially expressed than those without. Of the three remaining comparisons, two corresponded to comparisons between HO_5096_0412 and S0385 ($p = 0.17$, and $p = 0.055$), and one between HO_5096_0412 and Newman ($p = 0.054$). The second comparison between HO_5096_0412 and Newman was the most weakly significant result ($p = 0.025$). Thus, the two replicates for each individual pairwise comparison were largely concordant with each other.

Our analysis confirms that genes downstream of switched IGRs are on average more likely to be differentially expressed than genes not associated with IGR switches as identified using Piggy. To illustrate the genomic context and expression differences of genes with switched IGRs, we selected three of the most differentially expressed genes with IGR switches for the Newman vs MRSA252 comparison, and plotted nucleotide identity across the IGR (calculated as a 20-bp sliding window) alongside gene expression (Fig 4).

Compatibility and scalability

We have so far demonstrated that Piggy can be used to analyse the intergenic component of the pan-genome and identify IGR switches, and shown that these switches have biological relevance with respect to gene expression. Importantly, Piggy is designed such that the output files are compatible with existing software and databases. The “IGR_presence_absence.csv” file has an identical format to the “gene_presence_absence.csv” file produced by Roary, and can be loaded directly into the interactive browser-based viewer Phandango [33] (Fig S2). It can also be used as input, along with a traits file, to Scoary [34] to test for associations between

IGRs and phenotypic traits. Moreover, the “representative_clusters_merged.fasta” file can be loaded directly into BIGSdb [18] to create an allele scheme for IGRs. In order to provide proof-of-principle, we created a multilocus IGR (igMLST) scheme in BIGSdb. Briefly, 2,631 unique IGR sequences with length ≥ 30bp, from 7 *S. aureus* reference genomes, were entered into the database locus list. Using functionality within the database, these sequences were grouped as a searchable scheme (S_aureus_Intergenic_PIGGY), comparable to MLST, rMLST and wgMLST schemes [35–37]. The distribution of IGRs was analysed for all isolates in the database, identifying IGRs as present in the respective genome if a hit was recorded with nucleotide identity ≥ 70% over ≥ 50% of the sequence using a BLAST word size of 7 bp. The scheme can be found at [38]. Although we do not expect a typing scheme based solely on IGRs to be widely used, supplementing protein-coding regions with IGR alleles may provide additional information regarding links between genotype and phenotype, as well as increased epidemiological and phylogenetic resolution.

Discussion

Whole-genome sequence datasets consisting of hundreds or even thousands of bacterial isolates have revealed pan-genomes of many thousands of genes and large differences in gene content between isolates of the same species. Currently, pan-genome diversity is considered almost exclusively in terms of protein-coding genes, despite overwhelming evidence that variation within IGRs impacts on phenotypes. Here we address this by introducing Piggy, a pipeline specifically designed to incorporate IGRs into routine pan-genome analyses by working in close conjunction with Roary [4].

The utility of this approach is demonstrated using large datasets of *S. aureus* and *E. coli* ST131. Consistent with previous analyses of protein-coding regions [6,32], the IGR component of the ST131 pan-genome is considerably larger than that for ST22, and surprisingly is also larger than the pan-genome of the whole *S. aureus* species. There was more diversity within IGRs than genes in both species. While some IGRs may be essential for expression of multiple genes, IGRs are broadly subject to weaker purifying selection than protein coding genes [14]. The maintenance of core IGRs in both bacterial genome datasets is consistent with selection acting to conserve them and allows alignment and analysis in much the same way as protein-coding regions.

The current exclusion of IGRs from routine pan-genome or cgMLST analyses may in part reflect perceived difficulties in the alignment and subsequent cluster definition, particularly if the sequences are very short. We therefore validated the pipeline by investigating clustering accuracy as a function of sequence length by truncating the IGR sequences and recording whether they remained in the same cluster as their full-length counterparts. For *S. aureus*, the data showed that truncated IGRs > 20 bp almost always remained in the original cluster, confirming that the minimum length permitted in the pipeline of 30-bp is conservative. For *E. coli*, truncating the sequences had greater impact on cluster assignments, and a minimum length of 50 bp would be a safer setting in this case. The problems with clustering shorter sequences in *E. coli*, compared to *S. aureus*, are not due to the length of the sequence per se but reflect the higher rate of horizontal gene transfer in this species. This means that the IGRs are more likely to be chimeric in structure, with localised regions within the IGRs showing a high level of homology to different clusters. This lead to cluster assignment being dependant not so much on length, but on which part of the truncated sequence happened to be retained.

Variation within regulatory elements located within IGRs can impact on the expression of the downstream gene [17]. Piggy (alongside Roary) provides the means to combine information on genes and their cognate IGRs thus facilitating the detection of “switched” IGRs and downstream genes that are potentially affected. We have shown that in *S. aureus*, genes with switched upstream IGRs show a higher degree of differential expression than those without. This is consistent with previous work on *E. coli* [17], and suggests that the identification of IGR switches using Piggy can provide a useful indication of differential gene expression, even in the absence of RNA-seq data. However, we note that high divergence within IGRs does not necessarily imply selection for differential gene expression, and may instead simply reflect weaker selective constraints. A clear direction for future work is to make constructs consisting of genes with alternative IGRs, in order to directly measure the effect of natural IGR variants on gene expression. Similar experiments have previously been performed in *E. coli* based on variation within promoters [39], and IGRs more broadly [17]. The importance of changes in gene expression mediated by intergenic variation as a route of adaptation is currently unknown, but one recent study suggested that intergenic changes are strongly positively selected in *Pseudomonas aeruginosa* during infection in patients with cystic fibrosis, and more work is required to test the generality of these findings [16].

Conclusions

Driven by recent technical advances in high-throughput sequencing, large whole-genome datasets have provided powerful evidence concerning the genetic determinants that underlie complex multifactorial phenotypes such as virulence. Moreover, associating variation in core and accessory genes with phenotype data is providing new fundamental insight into the ecology and evolution of bacteria. However, in much the same way that non-protein coding DNA in the human genome was initially dismissed as “junk”, omitting IGRs from bacterial genome analysis severely limits our ability to draw inferences on the regulation of gene expression and associated phenotypic consequences. By developing Piggy as an easy-to-use bioinformatics tool with output files that are compatible with existing software and databases (eg Roary, Phandango; Figure S1, Scoary, BIGSdb) we envisage that combined information from genes and their cognate IGRs will vastly improve our understanding of genome evolution in bacteria.

Availability of supporting source code

Project name: Piggy

Project home page: https://github.com/harry-thorpe/piggy

Operating system(s): Linux

Programming language: Perl, R

Other requirements: Roary

License: GPLv3

RRID: (Piggy, RRID:SCR_015941)

Availability of supporting data

The *S. aureus* dataset was assembled from published genome sequences [19] available from the European Nucleotide Archive (ENA), study number ERP001012. The *S. aureus* RNA-seq data was previously published [20], and is available from the ENA, study number ERP009279. This was supplemented with the corresponding reference genomes, HO_5096_0412: HE681097, MRSA252: BX571856, Newman: AP009351, S0385: AM990992, available from the National Center for Biotechnology Information (NCBI). The *E. coli* ST131 dataset was from a previously published study [6], and is available at [21]. An archival copy of the Piggy source code is available via the *GigaScience* repository GigaDB[40].

Declarations

Ethics approval and consent to participate: Not applicable

Consent for publication: Not applicable

Competing interests: Not applicable

Funding: The *Staphylococcus aureus* genome sequences were generated as part of a study supported by a grant from the United Kingdom Clinical Research Collaboration Translational Infection Research Initiative and the Medical Research Council (grant number G1000803, held by Sharon Peacock) with contributions from the Biotechnology and Biological Sciences Research Council; the National Institute for Health Research on behalf of the Department of Health; and the Chief Scientist Office of the Scottish Government Health Directorate, on which E.J.F. was a principal investigator and S.C.B. was a postdoctoral researcher. H.A.T. is funded by a University of Bath research studentship. The funders had no role in study design, data collection and analysis, decision to publish, or preparation of the manuscript.

Authors' contributions: HAT designed and implemented the pipeline, and carried out the majority of the analyses, with input from EJF, SCB and SKS. HAT and EJF wrote the manuscript with input from SKS and SCB.

Acknowledgements: We are very grateful to Torsten Seemann, Andrew Page and João Carriço for encouragement and helpful feedback. We are also grateful to Matt Holden for provision of the *S. aureus* RNA-seq data, to Sandra Reuter for help with the *S. aureus* data, and to Alan McNally for the *E. coli* ST131 data. This work also greatly benefitted from access to the Medical Research Council funded Cloud Infrastructure for Microbial Bioinformatics (MRC-CLIMB) [41].

References

1. McInerney JO, McNally A, O'Connell MJ. Why prokaryotes have pangenomes. Nat Microbiol. 2017;2:17040.

2. Andreani NA, Hesse E, Vos M. Prokaryote genome fluidity is dependent on effective population size. ISME J. 2017;11:1719–21.

3. Medini D, Donati C, Tettelin H, Masignani V, Rappuoli R. The microbial pan-genome. Curr. Opin. Genet. Dev. 2005;15:589–94.

4. Page AJ, Cummins CA, Hunt M, Wong VK, Reuter S, Holden MTG, et al. Roary: rapid large-scale prokaryote pan genome analysis. Bioinformatics. 2015;31:3691–3.

5. Holt KE, Wertheim H, Zadoks RN, Baker S, Whitehouse CA, Dance D, et al. Genomic analysis of diversity, population structure, virulence, and antimicrobial resistance in Klebsiella

pneumoniae, an urgent threat to public health. Proc. Natl. Acad. Sci. U. S. A. 2015;112:E3574–81.

6. McNally A, Oren Y, Kelly D, Pascoe B, Dunn S, Sreecharan T, et al. Combined Analysis of Variation in Core, Accessory and Regulatory Genome Regions Provides a Super-Resolution View into the Evolution of Bacterial Populations. PLoS Genet. 2016;12:e1006280.

7. Vos M, Hesselman MC, te Beek TA, van Passel MWJ, Eyre-Walker A. Rates of Lateral Gene Transfer in Prokaryotes: High but Why? Trends Microbiol. 2015;23:598–605.

8. Fouts DE, Brinkac L, Beck E, Inman J, Sutton G. PanOCT: automated clustering of orthologs using conserved gene neighborhood for pan-genomic analysis of bacterial strains and closely related species. Nucleic Acids Res. 2012;40:e172.

9. Zhao Y, Wu J, Yang J, Sun S, Xiao J, Yu J. PGAP: pan-genomes analysis pipeline. Bioinformatics. 2012;28:416–8.

10. Sahl JW, Caporaso JG, Rasko DA, Keim P. The large-scale blast score ratio (LS-BSR) pipeline: a method to rapidly compare genetic content between bacterial genomes. PeerJ. 2014;2:e332.

11. Seemann T. Prokka: rapid prokaryotic genome annotation. Bioinformatics. 2014;30:2068–9.

12. Ochman H, Caro-Quintero A. Genome Size and Structure, Bacterial. In: Kliman RM, editor. Encyclopedia of Evolutionary Biology. Oxford: Academic Press; 2016. p. 179–85.

13. McCutcheon JP, Moran NA. Extreme genome reduction in symbiotic bacteria. Nat. Rev. Microbiol. 2011;10:13–26.

14. Thorpe HA, Bayliss SC, Hurst LD, Feil EJ. Comparative Analyses of Selection Operating on Non-translated Intergenic Regions of Diverse Bacterial Species. Genetics [Internet]. 2017; Available from: http://dx.doi.org/10.1534/genetics.116.195784

15. Molina N, Van Nimwegen E. Universal patterns of purifying selection at noncoding positions in bacteria. Genome Res. 2008;18:148–60.

16. Khademi H, Jelsbak L. Host adaptation mediated by intergenic evolution in a bacterial pathogen [Internet]. bioRxiv. 2017 [cited 2017 Dec 20]. p. 236000. Available from: https://www.biorxiv.org/content/early/2017/12/19/236000.article-info

17. Oren Y, Smith MB, Johns NI, Kaplan Zeevi M, Biran D, Ron EZ, et al. Transfer of noncoding DNA drives regulatory rewiring in bacteria. Proc. Natl. Acad. Sci. U. S. A. 2014;111:16112–7.

18. Jolley KA, Maiden MCJ. BIGSdb: Scalable analysis of bacterial genome variation at the population level. BMC Bioinformatics. 2010;11:595.

19. Reuter S, Török EM, Holden MTG, Reynolds R, Raven KE, Blane B, et al. Building a genomic framework for prospective MRSA surveillance in the United Kingdom and the Republic of Ireland. Genome Res. [Internet]. 2015; Available from: http://genome.cshlp.org/content/early/2015/12/15/gr.196709.115.abstract

20. Warne B, Harkins CP, Harris SR, Vatsiou A, Stanley-Wall N, Parkhill J, et al. The Ess/Type VII secretion system of Staphylococcus aureus shows unexpected genetic diversity. BMC

Genomics. 2016;17:222.

21. McNally et al. 2016 data repository [Internet]. Dryad. [cited 2016 Oct 25]. Available from: http://datadryad.org/resource/doi:10.5061/dryad.d7d71

22. Katoh K, Standley DM. MAFFT multiple sequence alignment software version 7: improvements in performance and usability. Mol. Biol. Evol. 2013;30:772–80.

23. Bray NL, Pimentel H, Melsted P, Pachter L. Near-optimal probabilistic RNA-seq quantification. Nat. Biotechnol. 2016;34:525–7.

24. Pimentel H, Bray NL, Puente S, Melsted P, Pachter L. Differential analysis of RNA-seq incorporating quantification uncertainty. Nat. Methods. 2017;14:687–90.

25. Tjaden B. De novo assembly of bacterial transcriptomes from RNA-seq data. Genome Biol. 2015;16:1.

26. RDevelopment CORE TEAM R, Others. R: A language and environment for statistical computing [Internet]. R foundation for statistical computing Vienna, Austria; 2008. Available from: http://www.R-project.org

27. Wickham H. Ggplot2: Elegant Graphics for Data Analysis. 2nd ed. Springer Publishing Company, Incorporated; 2009.

28. Fu L, Niu B, Zhu Z, Wu S, Li W. CD-HIT: accelerated for clustering the next-generation sequencing data. Bioinformatics. 2012;28:3150–2.

29. Camacho C, Coulouris G, Avagyan V, Ma N, Papadopoulos J, Bealer K, et al. BLAST+: architecture and applications. BMC Bioinformatics. 2009;10:421.

30. Feil EJ, Cooper JE, Grundmann H, Robinson DA, Enright MC, Berendt T, et al. How clonal is Staphylococcus aureus? J. Bacteriol. 2003;185:3307–16.

31. Lindsay JA, Holden MTG. Staphylococcus aureus: superbug, super genome? Trends Microbiol. 2004;12:378–85.

32. Holden MTG, Hsu L-Y, Kurt K, Weinert LA, Mather AE, Harris SR, et al. A genomic portrait of the emergence, evolution, and global spread of a methicillin-resistant Staphylococcus aureus pandemic. Genome Res. 2013;23:653–64.

33. Hadfield J, Croucher NJ, Goater RJ, Abudahab K, Aanensen DM, Harris SR. Phandango: an interactive viewer for bacterial population genomics. Bioinformatics [Internet]. 2017; Available from: http://dx.doi.org/10.1093/bioinformatics/btx610

34. Brynildsrud O, Bohlin J, Scheffer L, Eldholm V. Rapid scoring of genes in microbial pan-genome-wide association studies with Scoary. Genome Biol. 2016;17:238.

35. Maiden MCJ, Jansen van Rensburg MJ, Bray JE, Earle SG, Ford SA, Jolley KA, et al. MLST revisited: the gene-by-gene approach to bacterial genomics. Nat. Rev. Microbiol. 2013;11:728–36.

36. Jolley KA, Bliss CM, Bennett JS, Bratcher HB, Brehony C, Colles FM, et al. Ribosomal multilocus sequence typing: universal characterization of bacteria from domain to strain.

Microbiology. 2012;158:1005–15.

37. Sheppard SK, Jolley KA, Maiden MCJ. A gene-by-gene approach to bacterial population genomics: Whole genome MLST of Campylobacter. Genes . 2012;3:261–77.

38. Sheppard lab resources [Internet]. [cited 2018 Jan 16]. Available from: https://sheppardlab.com/resources

39. Shimada T, Yamazaki Y, Tanaka K, Ishihama A. The whole set of constitutive promoters recognized by RNA polymerase RpoD holoenzyme of Escherichia coli. PLoS One. 2014;9:e90447.

40. Thorpe HA, Bayliss SC, Sheppard SK, Feil EJ. Supporting data for ‘Piggy: A Rapid, Large-Scale Pan-Genome Analysis Tool for Intergenic Regions in Bacteria’. GigaScience database 2018. http://dx.doi.org/10.5524/100410

41. Connor TR, Loman NJ, Thompson S, Smith A, Southgate J, Poplawski R, et al. CLIMB (the Cloud Infrastructure for Microbial Bioinformatics): an online resource for the medical microbiology community. Microbial Genomics [Internet]. Microbiology Society; 2016 [cited 2016 Nov 1];2. Available from: http://mgen.microbiologyresearch.org/content/journal/mgen/10.1099/mgen.0.000086

Figure legends

Fig 1: An overview of the Piggy pipeline. a) A schematic to illustrate the Piggy pipeline and how it works alongside Roary [4]. b) IGRs are named according to their flanking genes and their orientations (CO_F - Co-Oriented Forward, CO_R - Co-Oriented Reverse, DP - Double Promoter, DT - Double Terminator). This naming scheme enables Piggy to link genes with their associated IGRs, and provides information on their orientations. c) A schematic to illustrate the difference between the "gene-pair" and "upstream" methods used to identify candidate switched IGRs. For the "gene-pair" method, only the IGR between the two genes is non-homologous ("switched"), and for the "upstream" method both the upstream IGR and gene may be non-homologous to the downstream gene.

Fig 2: Properties of the pan-genomes. Genes (red) and IGRs (blue) were analysed with frequency histograms (the number of genes/IGRs present in any given number of isolates). The vast majority of genes / IGRs are either very rare or very common. a) *S. aureus* ST22 b) *S. aureus* c) *E. coli* ST131.

Fig 3: *S. aureus* gene expression data. Pairwise RNA-seq comparisons between four *S. aureus* isolates, where two biological replicates were used for each isolate. The top-left of the diagonal corresponds to comparisons between replicate 1 from different isolates (e.g. SO385 replicate 1 vs HO_5096_0412 replicate 1). The bottom-right of the diagonal corresponds to comparisons between replicate 2 from different isolates (e.g. SO385 replicate 2 vs HO_5096_0412 replicate 2). The diagonal corresponds to comparisons between the two biological replicates from the same isolate. 2094 core genes were analysed in each comparison, and tpm (Transcripts per Kilobase Million) was used to quantify expression. The genes were separated into two categories: Switched (red), and Not-switched (grey), based on their upstream IGRs. The R^2 value corresponds to all the genes. The P-value corresponds to a Monte Carlo permutation test comparing the residuals of the two groups of genes, where a significant score indicates that the genes downstream of switch IGRs are associated with a higher degree of differential expression (ie higher residuals).

Fig 4: A detailed view of the genomic neighbourhood and expression data for selected genes in Newman vs MRSA252. Nucleotide identity was calculated using a 20 bp sliding window across the IGR, and this is shown alongside the flanking genes in their correct

orientation (left). The corresponding expression data for the gene of interest was also shown (right), with the two boxplots per isolate corresponding to the two biological replicates. a) dapE b) ssaA_1 c) ytrA.

Fig. S1: Clustering performance. The clustering performance was assessed by truncating IGR sequences and reclustering them with the pool of original sequences. Truncated IGRs which were placed into the same cluster as their progenitor sequences were deemed to be correctly clustered. a) *S. aureus* b) *E. coli*.

Fig S2: The IGR pan-genome as visualised using Phandango. A neighbour-joining phylogenetic tree was imported into Phandango [33] alongside the IGR_presence_absence.csv file. Each row corresponds to an isolate, and each column corresponds to an IGR, with the IGRs ordered from the left in order of decreasing frequency within the sample. The line graph at the bottom shows the frequency of the IGRs within the sample. a) *S. aureus* ST22 b) *E. coli* ST131.

Click here to download Table Table_1.pdf

Sheet1

Species	Core genes	Core IGRs	Accessory genes	Accessory IGRs	Percentage core genes	Percentage core IGRs
S. aureus ST22	2409	1556	816	1543	95	95
S. aureus	2129	1134	3446	8033	85	69
E. coli ST131	3930	2296	8876	14133	84	77

Table 1

Sheet1

Species	Core gene, Core IGR	Core gene, Accessory I	Accessory gene, Core IGR
S. aureus ST22	99.5	0.5	7.4
S. aureus	92.9	7.1	3.2
E. coli ST131	97.9	2.1	2.7

Table 2

Accessory gene, Accessory IGR
92.6
96.8
97.3

Figure 1

Click here to download Figure figure_1.png

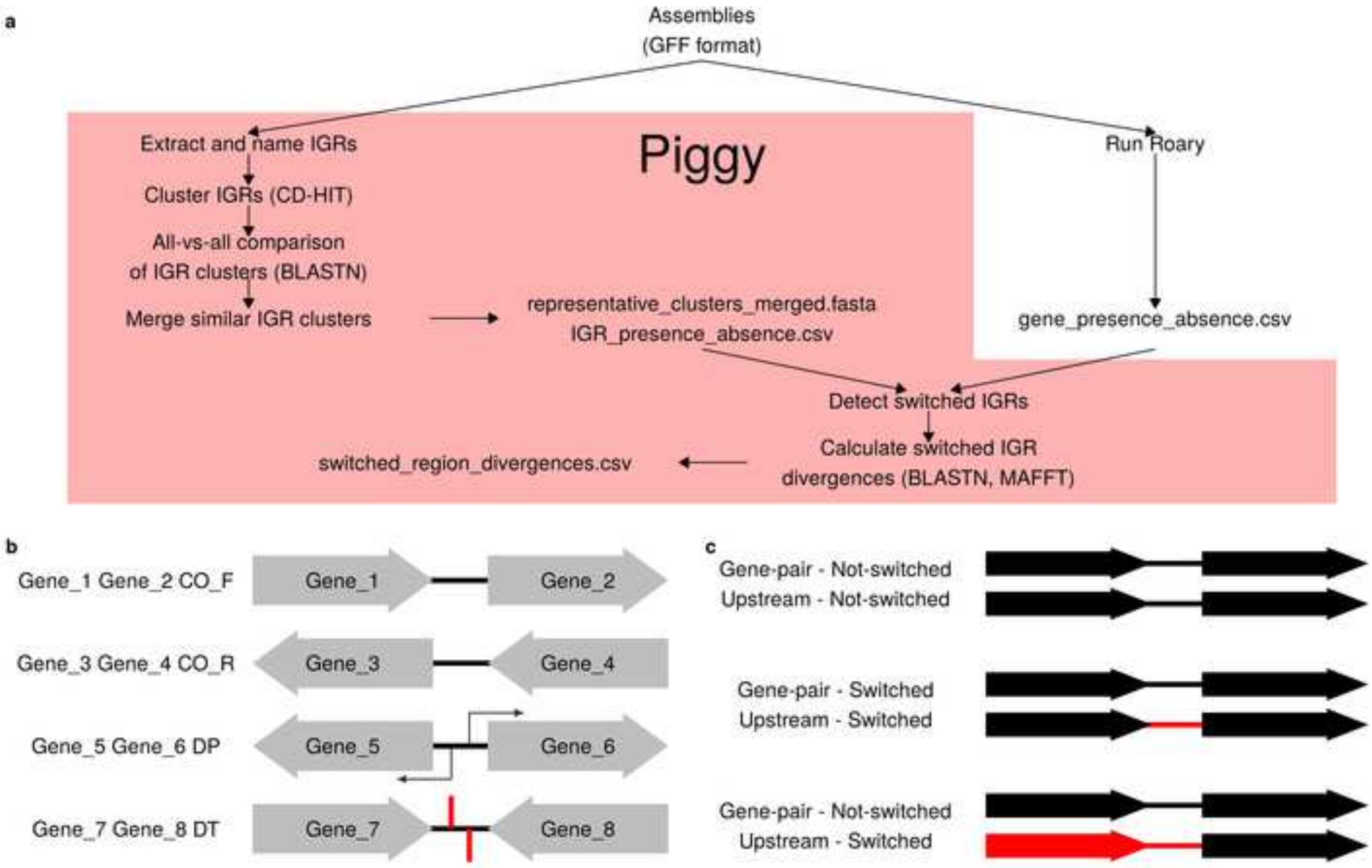

Figure 2

Click here to download Figure Figure_2.png

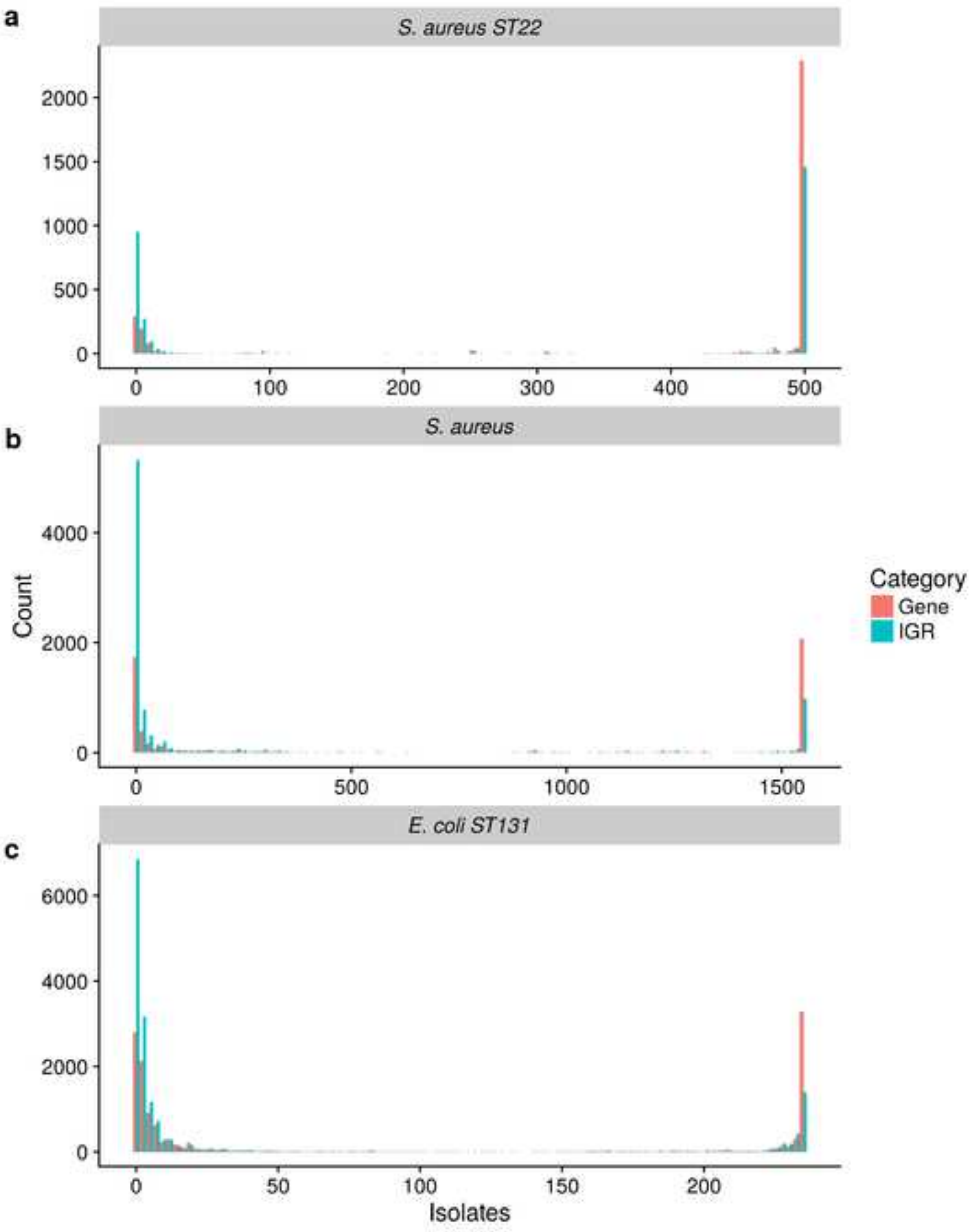

Figure 3

Click here to download Figure Figure_3.png

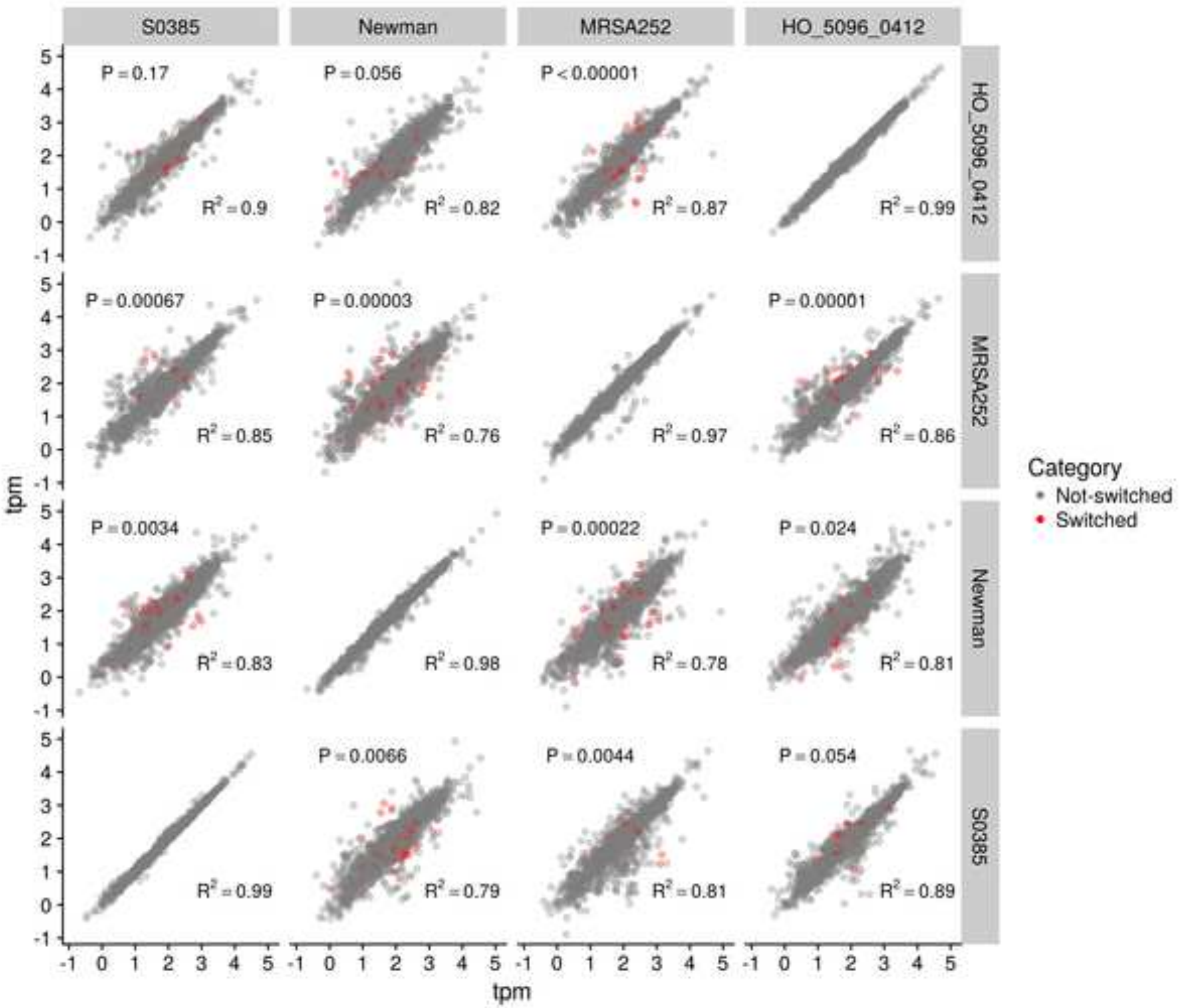

Figure 4

Click here to download Figure Figure_4.png

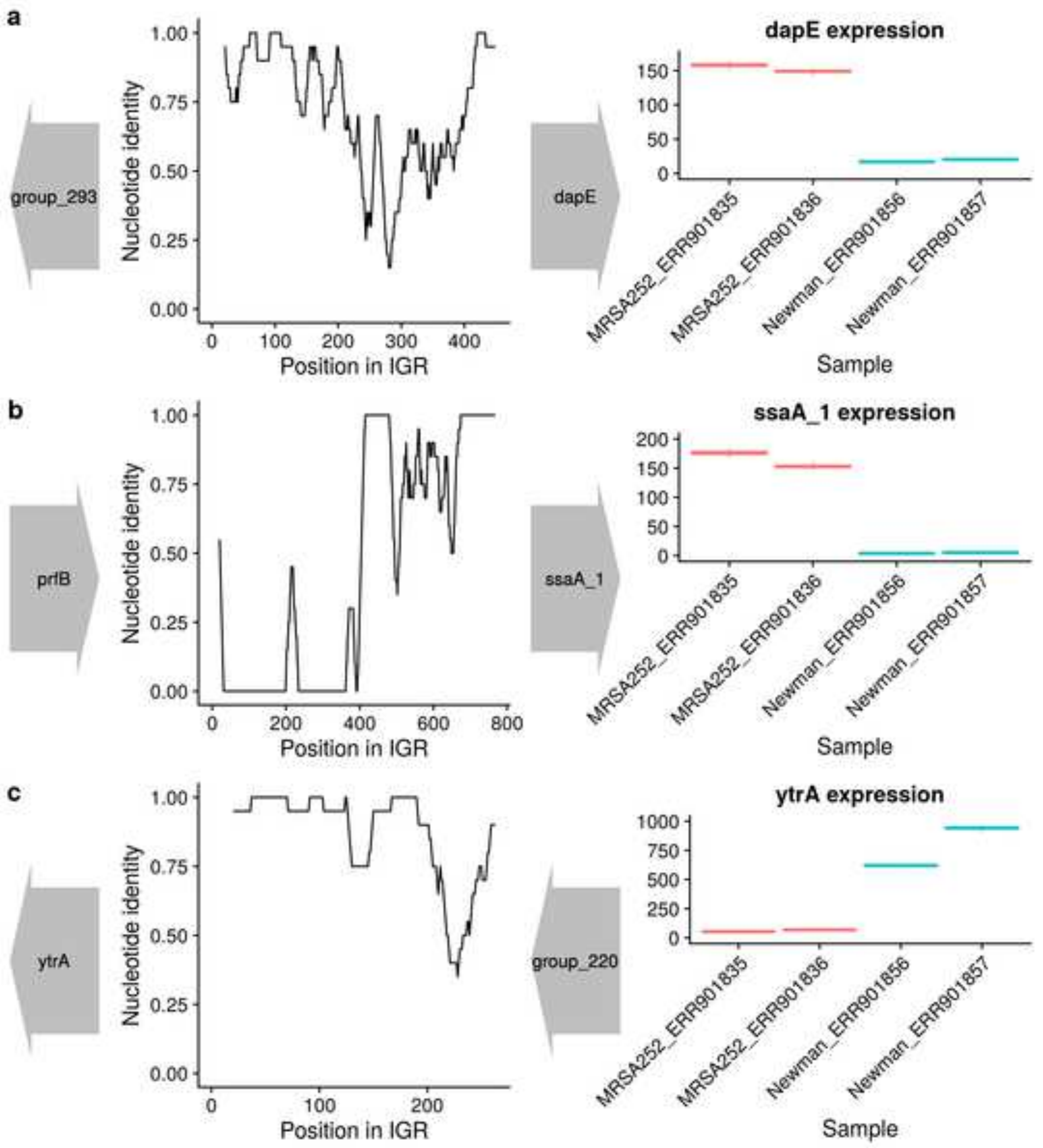

Supplementary Figure 2

Click here to access/download

Supplementary Material

Figure_S2.png

Supplementary Table 1

Click here to access/download
Supplementary Material
Table_S1.xlsx

Supplementary Figure 1

Click here to access/download
Supplementary Material
Figure_S1.png
