# Supplementary figures and images for: Piggy: a rapid, large-scale pan-genome analysis tool for intergenic regions in bacteria
Source: Gigascience. 2018 Mar 4;7(4):giy015. doi: 10.1093/gigascience/giy015 (PMC5890482; doi:10.1093/gigascience/giy015)

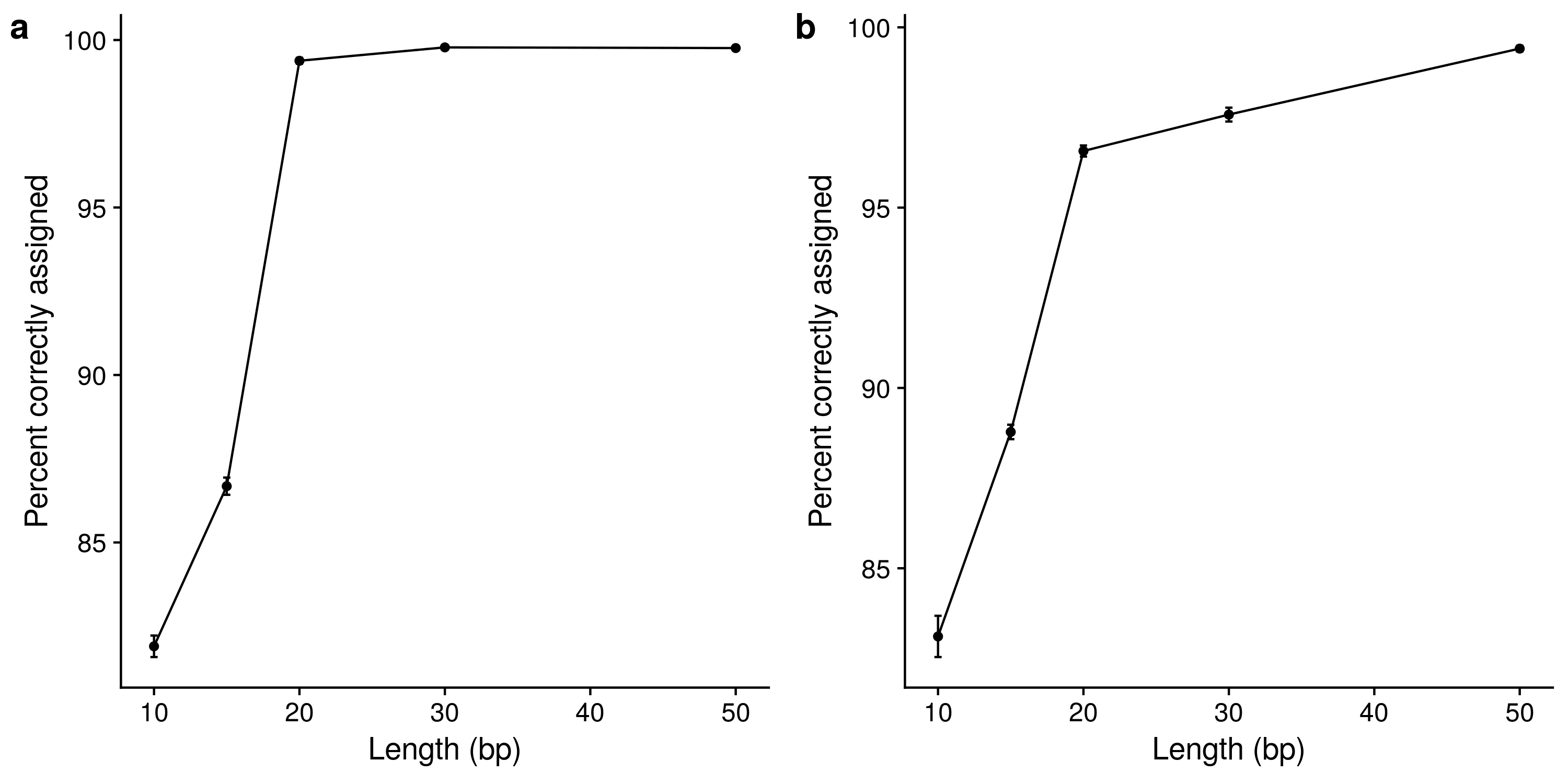

Supplement: Additional files [file giy015_supp.zip › Figure_S1.png]

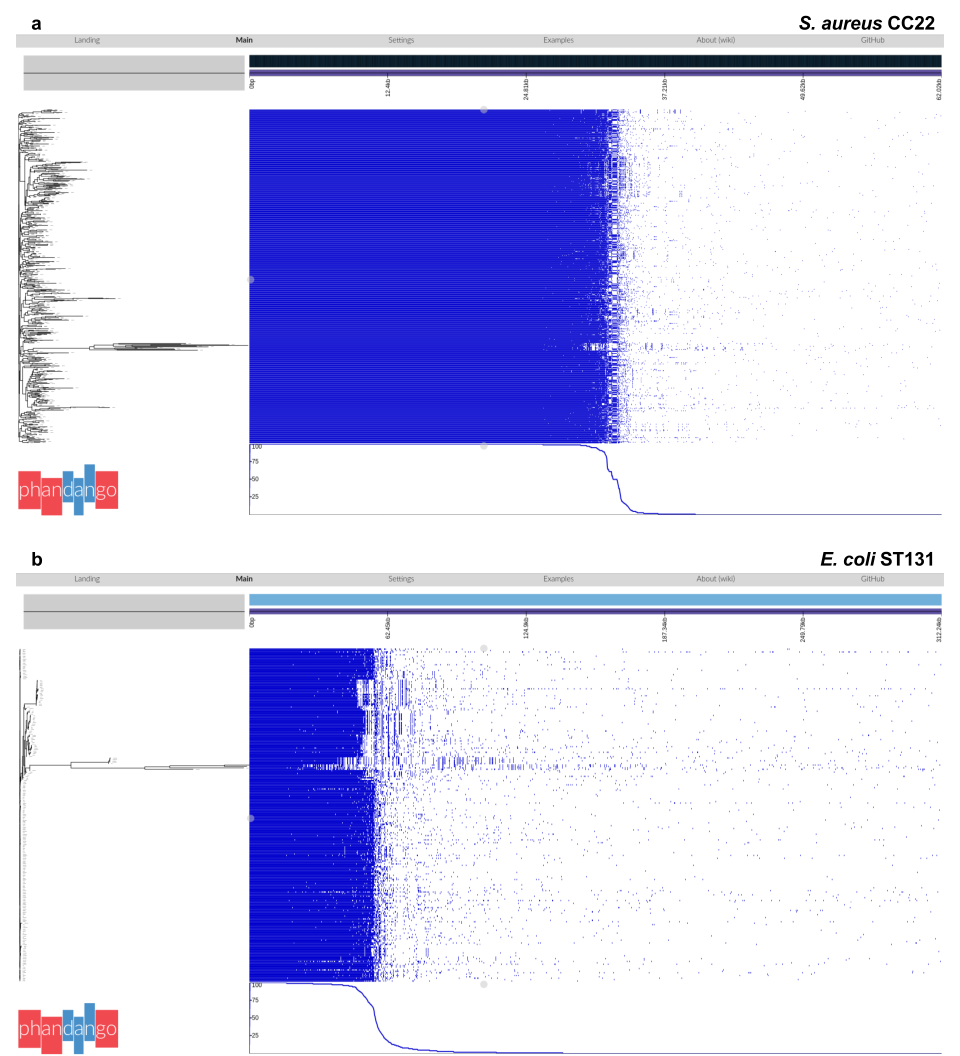

Supplement: Additional files [file giy015_supp.zip › Figure_S2.png]
