# Supplementary material for: Piggy: a rapid, large-scale pan-genome analysis tool for intergenic regions in bacteria
Source: Gigascience. 2018 Mar 4;7(4):giy015. doi: 10.1093/gigascience/giy015 (PMC5890482; doi:10.1093/gigascience/giy015)
Supplement: Response_to_Reviewer_Comments_Original_Submission.pdf [file giy015_response_to_reviewer_comments_original_submission.pdf]

Author's Response To Reviewer Comments

Reviewer reports:

Reviewer #1: Piggy represents a potentially valuable tool to the field of comparative genomics. In general, additional details on how the algorithm works would be helpful to understand the results.

RESPONSE: We thank the reviewer for recognizing the value of our approach – we have added additional details concerning the algorithm throughout the manuscript as requested.

P1,L16; bacteria "has" impacts

RESPONSE: P2,L35-36: This line now reads "variation in intergenic regions (IGRs) in bacteria can directly influence phenotypes"

P2.L9: Add references to this first line

RESPONSE: P2,L46: Added references: McInerney et al. 2017; Andreani et al. 2017

P2,L14: Relationship between pan-genome and core will differ greatly on the organism chosen

RESPONSE:We agree with this point and have added the following text:

P2,L59-62: "More generally, the relationship between the size of the core and accessory genomes varies between species. Broadly, ecological generalists have large accessory genomes, whilst more ecologically restricted species, such as endosymbionts, have much smaller accessory genomes (McInerney et al. 2017; Andreani et al. 2017)."

P2,L30-34: This is a run-on sentence and could be broken up to improve clarity

RESPONSE: P3,L67-70: This sentence now reads:

The increasing availability of datasets containing thousands of isolates thus offers an unprecedented opportunity for describing the genetic basis of bacterial adaptation, although the scale of these data presents serious logistic and conceptual challenges in terms of data management and analysis.

P3,L11: I have several problems with this statement about LS-BSR. What do you mean that it is no longer specific. Specific to what? Also, you mention that this reduced specificity is a by-product of pre-clustering, but the next sentence indicates that Roary also uses pre-clustering. Why wouldn't that also affect the results?

RESPONSE:P3,L74-77: We apologise for the confusion, and on reflection agree with the referee that the text was not reflective of the relative performance of the two methods. We have changed the text accordingly.

P3,L16-17: You mention that Roary is "more accurate than LS-BSR" and this is likely based on one comparison in the Roary paper. This was the result of one simulated dataset, using an unknown version of USEARCH and unknown parameters for alignment. To be safe, if you want to still report these results, I would mention that Roary was more accurate than LS-BSR using one simulated dataset, although the details remain unclear. You could safely remove this statement and not detract from the rest of your manuscript.

RESPONSE:P3,L74-77: Again, we completely agree with the referee and have modified the text accordingly.

P3,L39: Reference for "15% of the genome" statement?

RESPONSE:P3,L86: Added references: Ochman and Caro-Quintero 2016; McCutcheon and Moran 2011

P13,L4-6: What lengths of IGRs do you consider? Is there a minimum length? What do you do at the beginning and ends of draft contigs? More detail here would be very helpful.

RESPONSE:We have provided more detail in the text as requested:

P7,L204-206: IGRs at the edge of contigs are excluded by default, but when they are included (using the --edges flag) the missing information is denoted by NA, for example ‘Gene_1 NA NA’.

P7,L207-209: By default, only IGRs between 30-1000 bp in length are included by Piggy, though these lengths can be user-defined using the --size flag (minimum length = 30 bp).

P13,L27: What BLASTN parameters do you use to merge similar clusters?

P7,L218-219: More detail provided: BLASTN defaults, except -word_size = 10

P13,L27: What thresholds do you decide on for presence/absence?

P7,L219-221: Thresholds are provided by --len_id and --nuc_id, and these are used to produce clusters. Once the clusters have been produced, the gene presence information is simply a matrix of these clusters vs strains.

Fig S1: These trees look to be unrooted, but am unsure of why

RESPONSE:The phandango tool provides a visual comparison between the relatedness based on core genome variation with differences in gene content. The use of an outgroup to root the tree is not required for this.

Reviewer #2: The manuscript entitled: "Piggy: A Rapid, Large-Scale Pan-Genome Analysis Tool for Intergenic Regions in Bacteria" introduces the pipeline Piggy for the analysis of intergenic

regions (IGRs). The authors correctly point out that current approaches in pan-genome analysis focus purely on genes. They present a pipeline to address the remaining parts of the genome. Based on published RNA-seq data the manuscript highlights that especially for the analysis of gene expression the state of the intergenic region can be relevant and should be considered carefully.
Since the presented pipeline equals to a great extent the approach of the software Roary, the main contribution of this work is the identification of switched IGRs. In particular, the handling of differently annotated gene borders is solved in a clever way.
So far no standard file format for pangenomic data has established but the output format of Roary can be used by a bunch of analysis and visualization tools (panX, Phandango, FriPan).
It is thus reasonable to use this format for the output of Piggy.
Since for large parts of the intergenic regions in bacteria the function is unknown and most of these regions are very short, I am not sure how accurate the reconstruction of the "panIGRome" by Piggy currently is (see point 1. below).
However, before I can recommend accepting the manuscript there are some further points I would like to see addressed by the authors.:

Major points:

1. Intergenic regions in bacteria are usually much shorter than protein-coding sequences. Thus the clustering of these regions is potentially more vulnerable to wrongly aligned short sequences. Please add a part on the clustering performance to the manuscript.

RESPONSE:We thank the referee for this important point, and have spent considerable time addressing this issue in detail. Additional analyses on clustering performance are incorporated in the text (in both the Methods, P6,178-187, Results, P8-9,L252-271, and Discussion, P14,L445-458) as described below, and we feel this significantly improves the paper.

Our approach to examining clustering performance was based on truncating IGRs and re-clustering them with the original set of IGRs. This was based on the logic that if the truncation had no effect (i.e. if the same clusters were recovered), then this provides reassurance that the clustering is not confounded by the length of the sequences, at least within the relevant parameters we are using.

This approach confirmed that 20-30 bp represents a minimum length for reliable clustering of IGRs for S. aureus, but possible slightly longer for E. coli. The incorrect clustering at these lengths was mostly driven by IGRs which are homologous to other IGRs over part, but not all of the sequence (as a result of rearrangements, HGT etc). In these cases when the IGR was truncated it could align equally well with multiple original IGR sequences, depending on which section of the sequence was retained during truncation. This may be a problem at the edge of contigs, but these IGRs are (now) removed by default (updated in the newest version of Piggy on GitHub) - P7,L204-206. Due to the high number of incorrectly clustered IGRs when truncated to 10 bp, we recommend that these sequences are not included in the analysis at all.

2. page 16 line 27-39. Why did you use two different clusterings? One very loose clustering for Fig 2 and 3 and one more rigid for the rest of the manuscript? I do not see the point of using two

different clusterings. Either two IGRs have the same origin or not. There should be an optimal value for --len_id where the clustering is close to the true relationship. And this one should be used for all subsequent analyses.

RESPONSE:With respect, we feel that there is no true --len_id which is appropriate for all situations, in the same way that there is no true --nuc_id. Of course it is true that either IGRs have the same origin or not, but when faced with real data the rules for assigning clusters are essentially pragmatic rather than grounded in biological certainties. Hence Piggy (and Roary, LS-BSR, PanOCT) use thresholds to define clusters. An IGR may acquire a deletion in one strain which means it is no longer the same length as the same IGR in other strains, despite sharing a common history.

The loose setting (--len_id 10) was used to enable a fair comparison with Roary results, where genes of different lengths are frequently clustered together. These can be the result of genuine truncations or assembly errors. Roary only requires that genes are >120 bp in length, and does not require genes to be similar in length in order to cluster together (fully explained on P5-6,L152-168). The stricter setting (--len_id 90) was used to detect switching, as this enables downstream filtering based on either length or nucleotide identity (P6,L166-168).

3. The text emphasizes that it is so far unknown whether genes and IGRs should be considered as independent or closely linked units. Likely this will depend on the context of the scientific question. Instead of separate genes g or IGRs i the set of both (i,g) can be considered. In this case one could get a first impression on the linkage of both. While the identification of switched IGRs in the manuscript uses the information of the flanking genes, I would have loved to read a bit more about this link in the two data examples. How many core genes are flanked by core IGRs? How many different genes can be found next to the same IGR and how many different IGR does a gene have? Even a first impression on these numbers would improve the quality of the manuscript.

RESPONSE:We agree that this is an important consideration, and so have done an analysis which is designed to be a first impression on these numbers. We analysed the number of core and accessory genes which are immediately upstream of core and accessory IGRs, and presented these data in a table (Table 2), and also in the text:

RESPONSE:P10,L302-312: We used the output of Piggy to investigate the degree of linkage between genes and IGRs. We identified all genomic loci consisting of an IGR flanked by two genes, and from these we identified all pairs of genes and IGRs where the IGR was upstream of the gene. We then grouped these according to whether the gene or IGR was core or accessory (Table 2). For the S. aureus ST22 data, 99.5% of core genes were immediately downstream of a core IGR, and 92.9% of the accessory genes were similarly downstream of an accessory IGR. When considering the wider S. aureus dataset the figures were similar; 92.6% of core genes were downstream of a core IGR, and 96.8% of accessory genes were downstream of an accessory IGR. Thus, the assignment of an IGR as core or accessory is strongly predictive of the corresponding assignment of the cognate downstream gene, which in turn points to strong background linkage between genes in IGRs in the genome.

P10,L324-327: There was tight linkage between genes and IGRs, with 97.9% of core genes being immediately downstream of core IGRs and 97.3% of accessory genes being similarly downstream of accessory IGRs; these results are consistent with those from S. aureus (Table 2).

In addition, please state how you proceeded with genes where a gene has an IGR > 30bp in one strain and an IGR < 30bp in another strain. Are those genes excluded from your analysis?

RESPONSE:When an IGR was > 30 bp in one strain and < 30 bp in another, then those sequences > 30 bp would be included and the others would not. This is because the IGRs are selected before the clustering is done, and so the relationships between these sequences is not known.

4. The pan-genome can be studied at all levels of divergence from the level of single lineages within pathogenic strains up to the level of all bacteria. Piggy has been demonstrated in two closely related datasets based on a single lineage from S. aureus and E. coli, respectively. I am wondering if this is the envisaged distance of genomes to analyze and whether the pipeline can be used on more diverse datasets. In the former case, the manuscript should state more precisely that piggy is intended only for closely related bacterial strains. In the latter case, I would like to see the addition of some further more distantly related strains of S. aureus and/or E. coli.

RESPONSE:We have now included an additional analysis consisting of a diverse collection of 1500 S. aureus isolates (P9,L294, Fig 2b). This clearly shows that the size of the species-wide S. aureus pan-genome is much greater than that of ST22 (fourfold increase in the number of accessory genes, and fivefold increase in accessory IGRs) (Table 1). There was also a corresponding decrease in the number of core elements, although this was much more modest. That Piggy identified >2000 core genes and >1000 core IGRs suggests that Piggy can cope with diverse datasets (Table 1).

5. paragraph starting at page 9 at line 44:
In this paragraph a resampling method is used to show that between certain strains of S. aureus genes linked to a switched IGR are on average more differentially expressed than other genes. While the resampling approach is appropriate to produce p-values in this setting, I do not understand how these p-values have been adjusted. The Benjamini-Hochberg method is usually not used to change p-values, and one has to choose an acceptable false discovery rate. Which FDR did you choose? In addition, the observations need to be independent, which is clearly not the case in the 12 pairwise comparisons.
I would recommend to either just show the simulated p-values and choose a level of significance below 0.05 or explain much more detailed what has been adjusted and why.
In addition, please stick to lowercase "p" for the p-value. Also in Figure 4.

RESPONSE:P12,L384-393: The p-values have been left unadjusted, and those < 0.05 were deemed significant. Lowercase p was used throughout. “Independently” has been removed from the text.

6. I understand that the data provided by Piggy can be directly used to create an allele scheme. But I do not see the benefit of creating an allele scheme for IGRs compared to the wgMLST

schemes. Could you please clarify how this scheme could be used and what would be the advantage compared to MLST, rMLST and wgMLST?

RESPONSE:The IGR scheme is not expected to be used in isolation, but rather can be combined with a scheme based on genes which may offer increased resolution in very closely related sets of strains. We have added some explanation of this:

P13,L421-424: "Although we do not expect a typing scheme based solely on IGRs to be widely used, supplementing protein-coding regions with IGR alleles may provide additional information regarding links between genotype and phenotype, as well as increased epidemiological and phylogenetic resolution."

Minor issues:

Please explain more clearly why IGRs < 30 bp are excluded. Is this due to problems with the clustering and how did you determine the border at 30 bp?

RESPONSE:The exclusion of IGRs <30 bp is a conservative threshold as evidenced by the clustering assessment as described above.

Figure 1: The text in the flow diagram should be much larger.

RESPONSE:We have increased the size of the text in this figure.

Figure 2: In my opinion accumulation curves in pan-genome studies are not very informative and could easily be replaced by a simple table with the average number per genome and the total number in the pan-genome. I suggest to replace Fig 2b and Fig 3b by such a table and use the opportunity to replace vague statements about the gradient and the plateau of the accumulation curve in the text. The accumulation curves could still appear in the supplemental material.

RESPONSE:Figures 2 and 3 have been merged into one (Figure 2), and the accumulation curves and vague statements have been removed. A new table (Table 1) has been created and the text adjusted.

Figure 4: You could highlight the points in Figure 4 corresponding to the genes from Figure 5

RESPONSE:Figure 5 only serves as an illustration of the data using some example genes. Highlighting these genes on Figure 4 may draw unnecessary attention to them, and this is not the message we are trying to convey, which is that there is a moderate and widespread effect of IGR divergence on gene expression which is not limited to a few hand-picked genes.
