# Supplementary material for: Piggy: a rapid, large-scale pan-genome analysis tool for intergenic regions in bacteria
Source: Gigascience. 2018 Mar 4;7(4):giy015. doi: 10.1093/gigascience/giy015 (PMC5890482; doi:10.1093/gigascience/giy015)
Supplement: Response_to_Reviewer_Comments_Revision_1.pdf [file giy015_response_to_reviewer_comments_revision_1.pdf]

Author's Response To Reviewer Comments

To the editor:

Thank you for accepting our manuscript for publication in GigaScience.

We do not feel that it is necessary to host the supporting data in GigaDB as all the data is already published and available at public repositories as described in the manuscript.

Although in principle Code Ocean looks to be valuable, we do not think it is appropriate for Piggy for a number of reasons. First, Piggy requires various dependencies (particularly Roary), which are not installed on Code Ocean. Second, the free plan only provides 5 GB of storage and 1 compute hour per month - these are not sufficient for any real analysis of whole-genome sequence data. We will however consider it in any future projects.

We have reformatted the manuscript as per the guidelines.

To the reviewer:

We thank the reviewer for their detailed, constructive review, which helped us to improve the manuscript.
