# Supplementary material for: Piggy: a rapid, large-scale pan-genome analysis tool for intergenic regions in bacteria
Source: Gigascience. 2018 Mar 4;7(4):giy015. doi: 10.1093/gigascience/giy015 (PMC5890482; doi:10.1093/gigascience/giy015)
Supplement: Reviewer_1_Report_(Original_Submission) -- Jason Sahl — 12 Oct 2017 Reviewed [file giy015_reviewer_1_report_(original_submission).pdf]

Reviewer Report

Title: Piggy: A Rapid, Large-Scale Pan-Genome Analysis Tool for Intergenic Regions in Bacteria

Version: Original Submission **Date:** 12 Oct 2017

Reviewer name: Jason Sahl

Reviewer Comments to Author:

Piggy represents a potentially valuable tool to the field of comparative genomics. In general, additional details on how the algorithm works would be helpful to understand the results.

Specific comments that may improve the manuscript (no page numbers and line numbers that don't line up makes this more difficult). My page numbering will start at the Abstract (Page 1):
P1,L16; bacteria "has" impacts
P2.L9: Add references to this first line
P2,L14: Relationship between pan-genome and core will differ greatly on the organism chosen
P2,L30-34: This is a run-on sentence and could be broken up to improve clarity
P3,L11: I have several problems with this statement about LS-BSR. What do you mean that it is no longer specific. Specific to what? Also, you mention that this reduced specificity is a by-product of pre-clustering, but the next sentence indicates that Roary also uses pre-clustering. Why wouldn't that also affect the results?
P3,L16-17: You mention that Roary is "more accurate than LS-BSR" and this is likely based on one comparison in the Roary paper. This was the result of one simulated dataset, using an unknown version of USEARCH and unknown parameters for alignment. To be safe, if you want to still report these results, I would mention that Roary was more accurate than LS-BSR using one simulated dataset, although the details remain unclear. You could safely remove this statement and not detract from the rest of your manuscript.
P3,L39: Reference for "15% of the genome" statement?
P13,L4-6: What lengths of IGRs do you consider? Is there a minimum length? What do you do at the beginning and ends of draft contigs? More detail here would be very helpful.
P13,L27: What BLASTN parameters do you use to merge similar clusters?
P13,L27: What thresholds do you decide on for presence/absence?
Fig S1: These trees look to be unrooted, but am unsure of why

Level of Interest

Please indicate how interesting you found the manuscript: An article of importance in its field

Quality of Written English

Please indicate the quality of language in the manuscript: Acceptable

Declaration of Competing Interests

Please complete a declaration of competing interests, considering the following questions:

- Have you in the past five years received reimbursements, fees, funding, or salary from an organisation that may in any way gain or lose financially from the publication of this manuscript, either now or in the future?
- Do you hold any stocks or shares in an organisation that may in any way gain or lose financially from the publication of this manuscript, either now or in the future?
- Do you hold or are you currently applying for any patents relating to the content of the manuscript?
- Have you received reimbursements, fees, funding, or salary from an organization that holds or has applied for patents relating to the content of the manuscript?
- Do you have any other financial competing interests?
- Do you have any non-financial competing interests in relation to this paper?

If you can answer no to all of the above, write 'I declare that I have no competing interests' below. If your reply is yes to any, please give details below.

I declare that I have no competing interests

I agree to the open peer review policy of the journal. I understand that my name will be included on my report to the authors and, if the manuscript is accepted for publication, my named report including any attachments I upload will be posted on the website along with the authors' responses. I agree for my report to be made available under an Open Access Creative Commons CC-BY license (http://creativecommons.org/licenses/by/4.0/). I understand that any comments which I do not wish to be included in my named report can be included as confidential comments to the editors, which will not be published.

I agree to the open peer review policy of the journal

To further support our reviewers, we have joined with Publons, where you can gain additional credit to further highlight your hard work (see: https://publons.com/journal/530/gigascience). On publication of this paper, your review will be automatically added to Publons, you can then choose whether or not to claim your Publons credit. I understand this statement.

Yes
