# Supplementary material for: Piggy: a rapid, large-scale pan-genome analysis tool for intergenic regions in bacteria
Source: Gigascience. 2018 Mar 4;7(4):giy015. doi: 10.1093/gigascience/giy015 (PMC5890482; doi:10.1093/gigascience/giy015)
Supplement: Reviewer_2_Report_(Original_Submission) -- Franz Baumdicker — 16 Oct 2017 Reviewed [file giy015_reviewer_2_report_(original_submission).pdf]

Reviewer Report

Title: Piggy: A Rapid, Large-Scale Pan-Genome Analysis Tool for Intergenic Regions in Bacteria

Version: Original Submission **Date:** 16 Oct 2017

Reviewer name: Franz Baumdicker

Reviewer Comments to Author:

The manuscript entitled: "Piggy: A Rapid, Large-Scale Pan-Genome Analysis Tool for Intergenic Regions in Bacteria" introduces the pipeline Piggy for the analysis of intergenic regions (IGRs). The authors correctly point out that current approaches in pan-genome analysis focus purely on genes. They present a pipeline to address the remaining parts of the genome.
Based on published RNA-seq data the manuscript highlights that especially for the analysis of gene expression the state of the intergenic region can be relevant and should be considered carefully.
Since the presented pipeline equals to a great extent the approach of the software Roary, the main contribution of this work is the identification of switched IGRs. In particular, the handling of differently annotated gene borders is solved in a clever way.
So far no standard file format for pangenomic data has established but the output format of Roary can be used by a bunch of analysis and visualization tools (panX, Phandango, FriPan).
It is thus reasonable to use this format for the output of Piggy.
Since for large parts of the intergenic regions in bacteria the function is unknown and most of these regions are very short, I am not sure how accurate the reconstruction of the "panIGRome" by Piggy currently is (see point 1. below).
However, before I can recommend accepting the manuscript there are some further points I would like to see addressed by the authors.:

Major points:

1. Intergenic regions in bacteria are usually much shorter than protein-coding sequences. Thus the clustering of these regions is potentially more vulnerable to wrongly aligned short sequences. Please add a part on the clustering performance to the manuscript.

2. page 16 line 27-39. Why did you use two different clusterings? One very loose clustering for Fig 2 and 3 and one more rigid for the rest of the manuscript? I do not see the point of using two different clusterings. Either two IGRs have the same origin or not. There should be an optimal value for --len_id where the clustering is close to the true relationship. And this one should be used for all subsequent analyses.

3. The text emphasizes that it is so far unknown whether genes and IGRs should be considered as independent or closely linked units. Likely this will depend on the context of the scientific question. Instead of separate genes g or IGRs i the set of both (i,g) can be considered. In this case one could get a

first impression on the linkage of both. While the identification of switched IGRs in the manuscript uses the information of the flanking genes, I would have loved to read a bit more about this link in the two data examples. How many core genes are flanked by core IGRs? How many different genes can be found next to the same IGR and how many different IGR does a gene have? Even a first impression on these numbers would improve the quality of the manuscript.
In addition, please state how you proceeded with genes where a gene has an IGR > 30bp in one strain and an IGR < 30bp in another strain. Are those genes excluded from your analysis?

4. The pan-genome can be studied at all levels of divergence from the level of single lineages within pathogenic strains up to the level of all bacteria. Piggy has been demonstrated in two closely related datasets based on a single lineage from S. aureus and E. coli, respectively. I am wondering if this is the envisaged distance of genomes to analyze and whether the pipeline can be used on more diverse datasets. In the former case, the manuscript should state more precisely that piggy is intended only for closely related bacterial strains. In the latter case, I would like to see the addition of some further more distantly related strains of S. aureus and/or E. coli.

5. paragraph starting at page 9 at line 44:
In this paragraph a resampling method is used to show that between certain strains of S. aureus genes linked to a switched IGR are on average more differentially expressed than other genes.
While the resampling approach is appropriate to produce p-values in this setting, I do not understand how these p-values have been adjusted. The Benjamini-Hochberg method is usually not used to change p-values, and one has to choose an acceptable false discovery rate. Which FDR did you choose? In addition, the observations need to be independent, which is clearly not the case in the 12 pairwise comparisons.
I would recommend to either just show the simulated p-values and choose a level of significance below 0.05 or explain much more detailed what has been adjusted and why.
In addition, please stick to lowercase "p" for the p-value. Also in Figure 4.

6. I understand that the data provided by Piggy can be directly used to create an allele scheme. But I do not see the benefit of creating an allele scheme for IGRs compared to the wgMLST schemes. Could you please clarify how this scheme could be used and what would be the advantage compared to MLST, rMLST and wgMLST?

Minor issues:

Please explain more clearly why IGRs < 30 bp are excluded. Is this due to problems with the clustering and how did you determine the border at 30 bp?

Figure 1: The text in the flow diagram should be much larger.

Figure 2: In my opinion accumulation curves in pan-genome studies are not very informative and could easily be replaced by a simple table with the average number per genome and the total number in the pan-genome. I suggest to replace Fig 2b and Fig 3b by such a table and use the opportunity to replace vague statements about the gradient and the plateau of the accumulation curve in the text. The accumulation curves could still appear in the supplemental material.

Figure 4: You could highlight the points in Figure 4 corresponding to the genes from Figure 5

Level of Interest

Please indicate how interesting you found the manuscript: An article whose findings are important to those with closely related research interests

Quality of Written English

Please indicate the quality of language in the manuscript: Acceptable

Declaration of Competing Interests

Please complete a declaration of competing interests, considering the following questions:

- Have you in the past five years received reimbursements, fees, funding, or salary from an organisation that may in any way gain or lose financially from the publication of this manuscript, either now or in the future?
- Do you hold any stocks or shares in an organisation that may in any way gain or lose financially from the publication of this manuscript, either now or in the future?
- Do you hold or are you currently applying for any patents relating to the content of the manuscript?
- Have you received reimbursements, fees, funding, or salary from an organization that holds or has applied for patents relating to the content of the manuscript?
- Do you have any other financial competing interests?
- Do you have any non-financial competing interests in relation to this paper?

If you can answer no to all of the above, write 'I declare that I have no competing interests' below. If your reply is yes to any, please give details below.

I declare that I have no competing interests

I agree to the open peer review policy of the journal. I understand that my name will be included on my report to the authors and, if the manuscript is accepted for publication, my named report including any attachments I upload will be posted on the website along with the authors' responses. I agree for my report to be made available under an Open Access Creative Commons CC-BY license (http://creativecommons.org/licenses/by/4.0/). I understand that any comments which I do not wish to be included in my named report can be included as confidential comments to the editors, which will not be published.

I agree to the open peer review policy of the journal

To further support our reviewers, we have joined with Publons, where you can gain additional credit to further highlight your hard work (see: https://publons.com/journal/530/gigascience). On publication of this paper, your review will be automatically added to Publons, you can then choose whether or not to claim your Publons credit. I understand this statement.

Yes
