# Supplementary material for: Piggy: a rapid, large-scale pan-genome analysis tool for intergenic regions in bacteria
Source: Gigascience. 2018 Mar 4;7(4):giy015. doi: 10.1093/gigascience/giy015 (PMC5890482; doi:10.1093/gigascience/giy015)
Supplement: Reviewer_2_Report_(Revision_1) -- Franz Baumdicker — 21 Jan 2018 Reviewed [file giy015_reviewer_2_report_(revision_1).pdf]

Reviewer Report

Title: Piggy: A Rapid, Large-Scale Pan-Genome Analysis Tool for Intergenic Regions in Bacteria

Version: Revision 1 **Date:** 21 Jan 2018

Reviewer name: Franz Baumdicker

Reviewer Comments to Author:

he revised manuscript has addressed all major points and I recommend to accept it for publication.

In particular, the authors have added significant parts on the examination of clustering performance and the linkage between genes and IGRs. In addition, a further dataset (S. aureus) has been added to the analysis. Regarding the usage of different clusterings with different values for --len_id or --nuc_id, I understand that a fair comparison with Roary can only be done with a very low --len_id. Nonetheless, there will always be an optimal value for --len_id that minimizes a corresponding error. Since you had two very different situations/errors you needed to use two different parameters. The authors have added more details to the paragraph on parameter settings, such that this point is now well explained.

Congratulations on the nice work you have done.

Level of Interest

Please indicate how interesting you found the manuscript: An article of importance in its field

Quality of Written English

Please indicate the quality of language in the manuscript: Acceptable

Declaration of Competing Interests

Please complete a declaration of competing interests, considering the following questions:

- Have you in the past five years received reimbursements, fees, funding, or salary from an organisation that may in any way gain or lose financially from the publication of this manuscript, either now or in the future?
- Do you hold any stocks or shares in an organisation that may in any way gain or lose financially from the publication of this manuscript, either now or in the future?
- Do you hold or are you currently applying for any patents relating to the content of the manuscript?

- Have you received reimbursements, fees, funding, or salary from an organization that holds or has applied for patents relating to the content of the manuscript?
- Do you have any other financial competing interests?
- Do you have any non-financial competing interests in relation to this paper?

If you can answer no to all of the above, write 'I declare that I have no competing interests' below. If your reply is yes to any, please give details below.

I declare that I have no competing interests

I agree to the open peer review policy of the journal. I understand that my name will be included on my report to the authors and, if the manuscript is accepted for publication, my named report including any attachments I upload will be posted on the website along with the authors' responses. I agree for my report to be made available under an Open Access Creative Commons CC-BY license (http://creativecommons.org/licenses/by/4.0/). I understand that any comments which I do not wish to be included in my named report can be included as confidential comments to the editors, which will not be published.

I agree to the open peer review policy of the journal

To further support our reviewers, we have joined with Publons, where you can gain additional credit to further highlight your hard work (see: https://publons.com/journal/530/gigascience). On publication of this paper, your review will be automatically added to Publons, you can then choose whether or not to claim your Publons credit. I understand this statement.

Yes
